# Dietary β-carotene improves the ovary development and antioxidant capacity of replacement gilts

**DOI:** 10.1186/s40104-025-01342-2

**Published:** 2026-02-07

**Authors:** Jingya Jiang, Langduan Chen, Weiying Ma, Tingting Wen, Rui Liu, Guiyan Chu, Xiangfang Zeng, Shiyan Qiao, Chuanjiang Cai

**Affiliations:** 1 College of Animal Science and Technology, Northwest A&F University, Yangling, 712100 China; 2 Xi’an Tiankang Feed Co., Ltd., Xi’an, 710000 China; 3 Animal Genetic Breeding and Reproduction Key Laboratory of Sichuan Province, Sichuan Animal Science Academy, Chengdu, 610066 China; 4 College of Animal Science and Technology, China Agricultural University, Beijing, 100193 China

**Keywords:** β-Carotene, Estradiol, Follicular development, Oxidative stress, Replacement gilts

## Abstract

**Background:**

β-Carotene exhibits distinct biological effects that enhance reproductive performance in mammals; however, the mechanisms underlying these effects remain poorly understood. This study aimed to evaluate the effect of β-carotene on ovarian development in replacement gilts and to investigate its potential mechanisms.

**Results:**

A total of 20 gilts, aged 130 d, were randomly assigned to control group or β-carotene group (β-C group, diet containing 10 mg/kg of β-carotene). Each group consisted of 10 replicates, with one gilt per replicate, over a 60-d trial. β-Carotene significantly increased the number of follicles measuring 2–5 mm in diameter, elevated estradiol concentrations in both blood and follicular fluid of replacement gilts (*P* < 0.05). Compared to the control group, the β-C group exhibited a significant increase in β-carotene concentration within ovarian follicular fluid (*P* < 0.05). Transcriptomic analysis of GCs revealed that β-carotene could significantly upregulated the expression of Forkhead Box L2 (FOXL2). When β-carotene and its metabolic product were administered to granulosa cells (GCs), validation of differentially expressed genes in the transcriptome suggests the possibility that β-carotene, rather than its metabolic product, is responsible for the upregulation of FOXL2 in ovarian GCs, which subsequently may regulate StAR and enhance estradiol synthesis. Furthermore, β-carotene is likely to promote lipolysis, providing essential substrates for estradiol and adenosine triphosphate (ATP) production. Concurrently, β-carotene appears to increase the activity of the antioxidant enzymes superoxide dismutase 1 (SOD1) and glutathione peroxidase 4 (GPX4) in gilts, thereby reducing reactive oxygen species (ROS) (*P* < 0.05) and maintaining redox balance.

**Conclusions:**

Our findings suggest that β-carotene could promote lipolysis, activate the FOXL2-StAR pathway to increase estradiol synthesis in GCs, and alleviate oxidative stress, thereby contributing to follicle development.

**Supplementary Information:**

The online version contains supplementary material available at 10.1186/s40104-025-01342-2.

## Background

The development of follicles in replacement sows significantly influences the future economic performance of pig farms [1, 2]. Healthy follicle development is central to reproductive efficiency, directly influencing litter size [3], sow utilization lifespan, and overall farming costs. To enhance the reproductive performance of replacement sows, various strategies have been employed, including genetic selection methods [4] and the establishment of digital records for estrus-ovulation management [5]. Recently, advancements in precise nutritional regulation methods have emerged, focusing on adjusting key nutrient supplies, such as modifying dietary energy levels [6], supplementing dietary fiber [7, 8], and applying functional additives [9].

β-Carotene is recognized as a functional additive. It is not only the most active precursor of vitamin A (VA) among carotenoids but also a prevalent antioxidant in human diets and tissues [10]. Animals are unable to synthesize VA independently and thus require exogenous supplementation; however, excessive intake of VA can result in toxicity [11]. Due to its highly regulated intestinal absorption, β-carotene serves as a safe source of VA. Extensive research has demonstrated that β-carotene, as a natural antioxidant, plays a crucial role in maintaining and protecting eye and skin health [12, 13]. Recently, an increasing number of studies have indicated that β-carotene may have unique roles in animal reproduction.

Research on the role of β-carotene in dairy cow reproduction has been conducted for many years [14]. However, research regarding the reproductive effects of β-carotene in female mammals other than cattle is limited. Recent studies indicate that dietary β-carotene supplementation may enhance the activity of the antioxidant enzyme glutathione peroxidase (GSH-Px) and increase the levels of the vasodilator nitric oxide by promoting maternal gut microbiota diversity, thereby improving fetal blood circulation [15]. Furthermore, β-carotene supplementation has been demonstrated to reduce postpartum uterine bleeding, inhibit the production of inflammatory cytokines such as TNF-α and IL-4, and positively influence reproductive performance and postpartum recovery [16]. Based on these findings, we hypothesize that β-carotene positively influences ovarian development and the onset of estrus in replacement gilts.

We supplemented the diet of replacement gilts with β-carotene to evaluate its potential positive effects on ovarian development and the initiation of estrus. Additionally, we inferred the possible mechanism of β-carotene based on phenotypic results and omics data. To further validate our mechanistic hypothesis, we conducted experiments in vitro granulosa cells (GCs). By comparing the effects of β-carotene with its metabolites on GCs in vitro, we aimed to clarify whether the observed positive effects of β-carotene on ovarian development are attributable to the compound itself or its metabolites.

## Materials and methods

### Experimental design and diets

The experiment was conducted at the Livestock and Poultry Ecological Farming Station of Northwest A&F University. A total of 20 gilts, aged 130 d, were selected and randomly divided into two treatment groups, with 10 replicates per group, each containing one gilt. The control group received a basal diet, while the β-C group was supplemented with 10 mg/kg of β-carotene in the basal diet, administered twice daily for 60 d. The composition and nutritional level of the basal diet are presented in Table 1. This diet was specifically formulated with broken rice as the primary ingredient to establish a low background level of intrinsic carotenoids. Thus, the β-carotene in the experimental diet was derived almost entirely from the supplemented additive. The β-carotene feed additive was obtained from Guangzhou Juyuan Biochemical Co., Ltd. (China). Based on the product's recommended dosage and existing scientific research [18, 19] regarding β-carotene supplementation in sows, we selected a dosage of 10 mg/kg for the feed additive. VA was added to the basal diet at 4,000 IU/kg to meet the nutritional requirement without introducing confounding effects from excessive VA.

**Table 1:** Composition and nutrient levels of the basal diet (as-fed basis), %

Item	Content
Ingredients	
Broken rice	62.10
Soybean meal	10.30
Wheat bran	15.00
Soybean hull	8.90
CaH_2_PO_4_	1.10
NaCl	0.40
Limestone	1.20
Premix^a^	1.00
Total	100.00
Nutrients level	
DE^b^, MJ/kg	12.77
Organic matter	88.60
Crude protein	15.24
Crude fat	2.22
Crude fiber	6.18
Calcium	0.66
Total phosphorus	0.57

*DE* Digestible energy

^a^The premix provided the following per kg of the diet: VA 4,000 IU, VD 800 IU, VE 40 IU, VK 2 mg, VB_1_ 2 mg, VB_2_ 5 mg, VB_6_ 3 mg, VB_12_ 5 mg, nicotinic acid 20 mg, pantothenic acid 15 mg, folacin 1.5 mg, Fe 95.2 mg, Cu 4.53 mg, Zn 36 mg, Mn 34.6 mg, I 0.034 mg

^b^The digestible energy (DE) was calculated according to the nutrient requirements of swine by NRC (2012) [17]

All gilts were limit-fed throughout the trial to ensure consistent nutrient intake. The daily feed allowance was progressively increased according to the following schedule: 2.0 kg from 1 to 15 d, 2.2 kg from 16 to 30 d, 2.5 kg from 31 to 45 d, and 3.0 kg from 46 to 60 d. The β-carotene was uniformly blended into the diet as part of a 1% premix. To ensure stability, the complete feed was stored in opaque, sealed bags under dark and dry conditions. Oestrus was monitored daily in the gilts during the trial. The assessment included observations for a red and swollen vulva, the presence of vulval mucus, and a positive standing reaction to the back-pressure test.

### Sample collection and processing

On the final day of the experiment, various biological samples were collected from the replacement gilts for subsequent experimental analysis, including blood, uterine tubes, ovaries, liver, muscle, back fat, abdominal fat, and visceral fat. Blood samples were centrifuged at 3,000 × *g* for 10 min, and the serum was separated and stored at −80 °C. The ovaries and uterine tubes on both sides were excised, and the weights of the ovaries, uterine tubes, and uterus were measured separately. Additionally, the lengths of the uterine tubes on both sides were recorded. One ovary was fixed in 4% paraformaldehyde for subsequent sectioning and follicle counting, while follicular fluid was extracted from the other ovary using a syringe, centrifuged at 1,000 × *g* for 8 min, and GCs were isolated for transcriptomic sequencing. The follicular fluid was stored at −80 °C for hormone measurement and other analyses.

Ovarian tissues were fixed, dehydrated, and embedded in paraffin. Paraffin blocks were serially sectioned at a thickness of 10 μm. To achieve an unbiased and efficient estimation of follicle numbers, a systematic random sampling method was employed. Specifically, every 10^th^ section was collected, resulting in a final sampling interval of 100 μm between analyzed slides. The sampled sections were subjected to standard hematoxylin and eosin (H&E) staining. Follicle counting was performed on one ovary per animal. All H&E-stained sections were digitally scanned using a slide scanner (Aperio AT2, Leica Biosystems, Wetzlar, Germany) for detailed analysis. Follicles were classified and counted according to the following morphological criteria: Primordial follicle: An oocyte surrounded by a single layer of flattened or cuboidal granulosa cells; Primary follicle: An oocyte surrounded by a single layer of cuboidal granulosa cells; Secondary follicle: An oocyte surrounded by two or more layers of granulosa cells, with no visible antrum; Antral follicle: A follicle containing a clearly visible fluid-filled antral cavity; Atretic follicle: A follicle displaying an irregular or fragmented oocyte, pyknotic granulosa cell nuclei, and/or a broken basement membrane; Corpus luteum: A solid mass of luteal cells in the ovarian cortex, devoid of an oocyte.

To avoid double-counting, only follicles where the oocyte nucleus was clearly visible in the section plane were enumerated. This principle ensures that each follicle is counted only once, as the oocyte nucleus appears in a limited number of consecutive sections. Furthermore, each counted follicle was marked on the digital scan to prevent duplicate counts within the same section.

In this study, the number of biological replicates (*n*) varies across different experiments, determined by the experimental design and stringent quality control standards. The initial experimental population consisted of 10 sows in each group. Due to the lack of synchronization in estrus among the experimental animals, they were in different estrus stages, leading to the exclusion of statistical outliers. For transcriptomics and metabolomics analyses, we selected sows at the same physiological stage of estrus (*n* = 4 for each group) to minimize interference from physiological fluctuations. In some molecular experiments, the sample size is dictated by the availability of high-quality materials that meet all required detection criteria. The specific *n* value for each experiment is indicated in the illustrations, and this layered approach enhances the robustness and clarity of the data across each analytical platform.

### Isolation and culture of porcine GCs

For the in vitro experiments, porcine GCs were isolated from ovaries that were obtained from a commercial slaughterhouse (Besun, Yangling, China). These ovaries were sourced from crossbred sows (approximately 150 d) that were unrelated to the animals used in the in vivo β-carotene feeding trial described earlier.

Briefly, intact and morphologically normal ovaries were collected and placed in sterile physiological saline solution containing 1% penicillin–streptomycin, maintained at 37 °C. The ovaries were transported to the laboratory within 1 h of collection. Healthy follicles are characterized by a pinkish hue and clear, translucent follicular fluid. The cumulus-oocyte complexes (COCs) are distinctly visible on the follicular wall, which displays a taut outer membrane and a pronounced vascular pattern on its surface. In contrast, atretic follicles appear pale grey or white, with COCs and numerous GCs shed into the follicular cavity. The follicular fluid becomes cloudy, and white debris may be observed deposited within the cavity. Follicular fluid from healthy antral follicles (2–5 mm in diameter) was aspirated using an 18-gauge needle and a 10-mL syringe. The aspirated fluid was centrifuged at 1,000 × *g* for 8 min to pellet the GCs. The cell pellet was washed twice with phosphate-buffered saline (PBS).

The harvested GCs were cultured in DMEM/F-12 medium (Gibco, New York, USA) supplemented with 10% fetal bovine serum (FBS, Gibco, New York, USA) and 1% penicillin–streptomycin at 37 °C in a humidified atmosphere with 5% CO_2_. The GCs were seeded at a density of 2 × 10^5^ cells/cm^2^ in culture plates and allowed to adhere for 24 h in a 5% CO_2_ incubator at 37 °C. After this adherence period, the old medium was discarded, and the cultures were gently rinsed once with PBS. Fresh medium was then added to remove non-adherent cells and debris. At this point, the cell density reached approximately 50% confluence. Only after this washing step were the cells subjected to the specified experimental treatments.

To analyze the baseline expression of markers (e.g., FOXL2) across follicle development, an additional set of GCs was collected directly from healthy follicles of specific size categories without in vitro culture. Ovaries were dissected, and follicles were carefully isolated and grouped into three categories based on their diameter: small follicles (0–2 mm), medium follicles (2–5 mm), and large follicles (> 5 mm). The cells from each size category were pooled separately and processed immediately for total RNA and protein extraction.

### CCK8

The concentrations of β-carotene (1.25, 2.5, 5, 10, 20, 40, 80 μmol/L), retinol, retinaldehyde, retinoic acid (RA) (1.25, 2.5, 5, 10 μmol/L) were selected based on literature-reported effective ranges for oocyte maturation (10 μmol/L) [20] and GC steroidogenesis (0.1–10 μmol/L) [21], respectively. The effects of varying concentrations of β-carotene, retinol, retinaldehyde, and RA on the viability of GCs were assessed using the Cell Counting Kit-8 (IV08, Invigentech, Xi'an, China). GCs were seeded into 96-well plates at a density of 2 × 10^5^ cells/cm^2^ (equivalent to 6 × 10^4^ cells/well) and washed with PBS after 24 h of culture. Subsequently, different concentrations of β-carotene, retinol, retinaldehyde, and RA were added to the 96-well plates and incubated for an additional 24 h. Following medium replacement, 10 μL of CCK solution was added to each well, and the plates were incubated for 1 h. Absorbance was measured at 450 nm using a microplate reader (Elx808/Elx808IU, BioTek, USA). Cell viability for each treatment concentration was calculated based on the absorbance values. Each treatment condition within an independent experiment was assessed in 3 or 4 technical replicates. The entire assay was independently repeated 4 times with cells from separate isolations.

### ELISAs

Estradiol and progesterone levels in follicular fluid and culture medium were quantified using Estradiol ELISA Kit (YJ002366) and the Progesterone ELISA Kit (YJ911258; Mlbio, Shanghai, China), following the manufacturers' protocols. Additionally, concentrations of β-carotene, retinol, retinaldehyde, and RA in serum and culture medium were assessed using the pig β-Carotene ELISA Kit (MB-200019A), the Retinol ELISA Kit (MB-10081A), the Retinaldehyde ELISA Kit (MB-10085A), and the RA ELISA Kit (MB-10089A; Jiangsu Meibiao Biotechnology, Jiangsu, China). Furthermore, reactive oxygen species (ROS) concentrations and antioxidant enzyme activities were measured using the ROS ELISA Kit (YX-181519P), the Superoxide Dismutase 1 (SOD1) ELISA Kit (YX-191505P), the Superoxide Dismutase 2 (SOD2) ELISA Kit (YX-191506P), the Glutathione Peroxidase 4 (GPX4) ELISA Kit (YX-071628P), and the Catalase (CAT) ELISA Kit (YX-030120P; Youxuan Biotechnology, Shanghai, China). All assay indicators in serum and follicular fluid were measured directly, as preliminary tests confirmed that the sample values were within the linear range of the standard curve.

### Transcriptome analysis

To further investigate the molecular mechanisms by which β-carotene affects GC function, we conducted transcriptome sequencing analysis. The transcriptome sequencing analysis was performed using *n* = 4 biologically independent granulosa cell samples per group. Initially, total RNA was isolated and purified from samples using TRIzol (15596018, Thermofisher, USA), and its integrity was verified to ensure all samples had an RNA Integrity Number (RIN) > 7.0. Strand-specific RNA-seq libraries were constructed using oligo(dT) beads for mRNA enrichment and the dUTP method, and were sequenced on an Illumina NovaSeq™ 6000 platform by LC Bio Technology Co., Ltd. (Hangzhou, China) to generate 150-bp paired-end (PE150) reads.

The raw sequencing data were preprocessed to remove adapters and low-quality sequences using FastQC, yielding high-quality clean data. The high-quality sequencing data were subsequently aligned to the pig reference genome (Sus scrofa, Ensembl release v107) using HISAT2 (v2.2.1), and gene expression quantification was performed using StringTie (v2.1.6) with FPKM as the normalized expression metric.

Differential gene expression between groups was analyzed using DESeq2 and edgeR software. Significantly differentially expressed genes (DEGs) were identified based on the thresholds of an absolute log_2_ fold change ≥ 1 and a false discovery rate (FDR) *q*-value < 0.05. Functional enrichment analysis, including Gene Ontology (GO) and Kyoto Encyclopedia of Genes and Genomes (KEGG) pathway analyses, was performed on the DEGs using the hypergeometric test. All data analysis and visualization were conducted using R software (version 3.6) on the OmicStudio platform.

### Untargeted metabolomics analysis

To further investigate the effects of β-carotene on pig follicular fluid metabolites, we conducted an untargeted metabolomics analysis. The untargeted metabolomics analysis was performed using *n* = 4 biologically independent follicular fluid samples per group. This analysis was performed at Wuhan Metviare Biotechnology Co., Ltd. (Wuhan, China), utilizing liquid chromatography-tandem mass spectrometry (LC–MS/MS) for the detection and screening of differential metabolites. Follicular fluid samples (50 μL) were subjected to protein precipitation with 150 μL of a 20% ethanol-methanol extraction solution containing internal standards, followed by vortexing and centrifugation to obtain the supernatant for LC–MS/MS analysis. The metabolites were separated and detected using an ultra-high-performance liquid chromatograph (LC-30A, Shimadzu, Japan) in conjunction with a mass spectrometer (TripleTOF 6600+, SCIEX, Foster City, CA, USA). Chromatographic separation was achieved on a Waters Acquity Premier HSS T3 Column (1.8 μm, 2.1 mm × 100 mm, Waters Corporation, USA) maintained at 40 °C, with a flow rate of 0.4 mL/min and an injection volume of 4 μL. The mobile phase consisted of 0.1% formic acid in water (A) and 0.1% formic acid in acetonitrile (B), using the following gradient: 5% B to 20% B over 2.0 min, 40% B at 5.0 min, 99% B at 6.0 min, held until 7.5 min, and returned to 5% B at 7.6 min for re-equilibration. A comprehensive quality control (QC) strategy was implemented, which included the regular injection of pooled QC samples. The stability of the analysis was monitored by overlaying total ion chromatograms of QC samples, assessing the high correlation between QC samples, and confirming the low coefficient of variation (CV < 0.3) of internal standards and the majority of metabolites. Metabolite identification confidence was ensured by filtering for metabolites with MS/MS spectral matches (Level 1 identification) and a comprehensive identification score above 0.5. Additionally, differential metabolites were identified through a combination of univariate and multivariate statistical analyses. Specifically, orthogonal projections to latent structures-discriminant analysis (OPLS-DA) was performed, and metabolites with a variable importance in projection (VIP) > 1 and a Student's *t*-test *P*-value < 0.05 were considered significantly altered and enriched metabolic pathways were identified.

### RNA extraction, reverse transcription, and quantitative PCR

Total RNA was isolated from primary porcine GCs using the AG RNAex Pro Reagent (AG21102, Accurate Biotechnology, Hunan, China) according to the manufacturer's protocol. RNA concentration and purity were determined spectrophotometrically, with samples having an A_260_/A_280_ ratio between 1.9 and 2.0 used for subsequent analysis.

For cDNA synthesis, 500 ng of total RNA was reverse transcribed using HiScript III RT SuperMix for qPCR (+ gDNA wiper) (R323, Vazyme, Nanjing, China) in a 10 µL reaction system, following the manufacturer's instructions. This kit includes a specific gDNA removal step that efficiently digests any residual genomic DNA prior to the reverse transcription reaction, ensuring that the resulting cDNA is free from DNA contamination.

Quantitative PCR (qPCR) was performed using ChamQ SYBR qPCR Master Mix (Q311, Vazyme, Nanjing, China) on a QuantStudio™ 3 Real-Time PCR System (Applied Biosystems, USA). Each 10 µL reaction contained 5.2 µL of ChamQ SYBR Master Mix, 0.2 µL of each forward and reverse primer (10 μmol/L), and 4.4 µL of cDNA template (diluted 1:15). The thermal cycling protocol included an initial denaturation at 95 °C for 3 min, followed by 35 cycles of denaturation at 95 °C for 10 s, and annealing/extension at 60 °C for 30 s. Fluorescence data were collected at the end of each annealing/extension step. After amplification, a melt curve analysis was performed by increasing the temperature from 55 °C to 95 °C in 0.15 °C increments to verify the specificity of the amplification products.

To ensure the reliability of the qPCR data, the following controls were included in each run: a no-template control (NTC) for each primer pair to detect potential reagent contamination, and a no-reverse transcription control (RT) to confirm the absence of genomic DNA amplification.

The primer sequences used in this study are listed in Table S1. All primers were designed based on mRNA sequences obtained from the NCBI database and, wherever possible, were designed to span an exon–exon junction to avoid amplification of genomic DNA. All primers—whether adopted from previous literature or newly designed in this study—were validated for performance. The specificity of each primer pair was confirmed by a single peak in the melt curve analysis.

Relative gene expression levels were calculated using the comparative 2^–∆∆Ct^ method, with porcine β-actin used as the reference gene for normalization, as its expression was stable across experimental groups.

### Protein extraction and Western blot

All Western blot experiments were independently repeated three times, each being performed on GCs cultured in 6-well plates and subjected to the specified treatments. Following treatment, the cells were washed twice with cold PBS and lysed directly in the wells using RIPA lysis buffer (P0013B, Beyotime, Shanghai, China) supplemented with 1 mmol/L PMSF (ST2573, Beyotime). The lysates were then centrifuged at 12,000 × *g* for 15 min at 4 °C, and the supernatants were collected. The protein concentration of each supernatant was determined using a BCA Protein Assay Kit (Enhanced; P0010, Beyotime) according to the manufacturer's instructions.

To ensure the specificity of the results, two types of controls were included in each experiment: omission of the primary antibody and negative control cellular samples. Protein samples were prepared by mixing the lysates with 5X SDS-PAGE Loading Buffer (LT103, Yeasen, Shanghai, China) at a 4:1 (v/v) ratio. The mixtures were denatured by boiling at 100 °C for 10 min. Equal amounts of total protein (20 μg) were loaded into each lane and separated by electrophoresis on 4%–12% SurePAGE gels (X15412Gel, ACE Biotechnology, Shanghai, China) at a constant voltage of 120 V. The separated proteins were subsequently transferred onto polyvinylidene fluoride (PVDF) membranes (E801-01, Vazyme, Nanjing, China) by electroblotting at a constant current of 200 mA.

The membranes were blocked for 1 h at room temperature using 5% (w/v) non-fat milk prepared in TBST (Tris-Buffered Saline with 0.1% Tween-20). After blocking, the membranes were incubated with the appropriate primary antibodies overnight at 4 °C. Following five washes (5 min each) with TBST, the membranes were incubated with the corresponding horseradish peroxidase (HRP)-conjugated secondary antibodies for 2 h at room temperature. After another five washes with TBST, protein bands were visualized using Immobilon Western Chemiluminescent HRP Substrate (WBKLS0500, Millipore, Germany) and captured using a chemiluminescence imaging system. Band intensities (gray values) were quantified using ImageJ software. The antibodies used in the Western blot analysis are listed in Table S2.

The acquired digital images were quantitatively analyzed using ImageJ. A rectangular selection tool was employed to define each protein band of interest. The integrated density, defined as the sum of the pixel values within the selected area, was measured for each band. Subsequently, an adjacent background area of identical size was selected, and its integrated density was measured. The background signal was subtracted from the corresponding band signal to obtain the final net intensity value for each band. To account for potential variations in protein loading and transfer efficiency, the net intensity of each target protein band was normalized to the net intensity of the β-actin band from the same sample/lane. The resulting normalized values (Target/β-actin) were utilized for all subsequent statistical analyses and graphical representations.

### Cell transfection

After 24 h of cell adhesion, the cells were washed with PBS, resulting in a cell density of approximately 50%. GCs were cotransfected with either targeting small interfering RNA (siRNA) using X-tremeGENE 360 Transfection Reagent (Roche, CH, Germany) or flag-pcDNA plasmid using X-tremeGENE HP DNA Transfection Reagent (Roche, CH, Germany) for a duration of 24 h. The sequence of the siRNA used is shown in Table S3.

### CUT&Tag-qPCR

CUT&Tag-qPCR was performed using the Hyperactive Universal CUT&Tag Assay Kit for Illumina Pro (Vazyme, Nanjing, China), following the manufacturer’s protocol with three independent biological replicates per condition. Briefly, GCs from both the control and β-C groups were digested with trypsin, and 100,000 cells per group were bound to ConA Beads Pro. The beads were then incubated overnight at 4 °C with FOXL2 primary antibody, followed by a 1-h incubation at room temperature with a secondary antibody. A parallel negative control, in which the primary antibody was omitted, was included in all experiments. Subsequently, pA/G-Tnp Pro was added and incubated for 1 h at room temperature. Tagmentation was induced by adding TTBL and incubating at 37 °C for 60 min in a thermal cycler. The reaction was stopped by adding 10% SDS and incubating at 55 °C for 10 min. DNA was extracted from the supernatant and subjected to qPCR analysis using the primers listed in Table S4. Significantly higher enrichment with the FOXL2 antibody versus the negative control demonstrated assay specificity.

### Oil Red O staining

To assess lipid accumulation in GCs following various treatments, the cells were fixed in a 24-well plate using Oil Red O fixative. Staining was conducted according to the instructions provided with the Oil Red O staining kit (G1262, Solarbio, Beijing, China), and images were captured using an inverted fluorescence microscope (IX73, Olympus, Tokyo, Japan). Lipid deposition was quantified by analyzing stained images using ImageJ software. The color images were converted to 8-bit grayscale, and a consistent threshold was applied to all images to highlight the stained lipid droplets. The total area of positive staining relative to the total area of each field of view was measured and calculated as a percentage.

### ATP production assay

To assess the effects of β-carotene and RA treatment on ATP production in GCs, we utilized the enhanced ATP detection kit (S0027, Beyotime, Shanghai, China) to quantify ATP production in GCs following various treatments, in accordance with the manufacturer's instructions. Measurements were conducted using a multifunctional microplate reader (Elx808/Elx808IU, BioTek, USA).

### Statistical analyses

All statistical analyses were conducted using GraphPad Prism 9. Data are presented as the mean ± standard error of the mean (SEM), with a significance threshold set at *P* < 0.05.

The experimental unit for in vivo studies was defined as the individual gilt. For in vitro cell culture experiments, the experimental unit was one biological replicate, defined as granulosa cells isolated from a distinct batch of porcine ovaries. Each experiment included a minimum of *n* = 3 independent biological replicates, with technical replicates (triplicate wells) used within each biological replicate to account for procedural variability. The exact sample size (*n*) for each experiment is provided in the corresponding figure legend.

Before parametric testing, all datasets were assessed for normality using the Shapiro–Wilk test and for homogeneity of variances using the Brown–Forsythe test. Datasets that met both assumptions were analyzed as follows: (1) Comparisons between two groups were performed using an unpaired, two-tailed Student’s *t*-test. (2) Comparisons among three or more groups were performed using one-way analysis of variance (ANOVA), followed by Tukey’s post hoc test for pairwise comparisons among all groups, or Dunnett’s post hoc test for comparisons against a single control group.

## Results

### β-Carotene supplementation increased the number of medium follicles and elevated estradiol concentrations in gilts

**Fig. 1:**
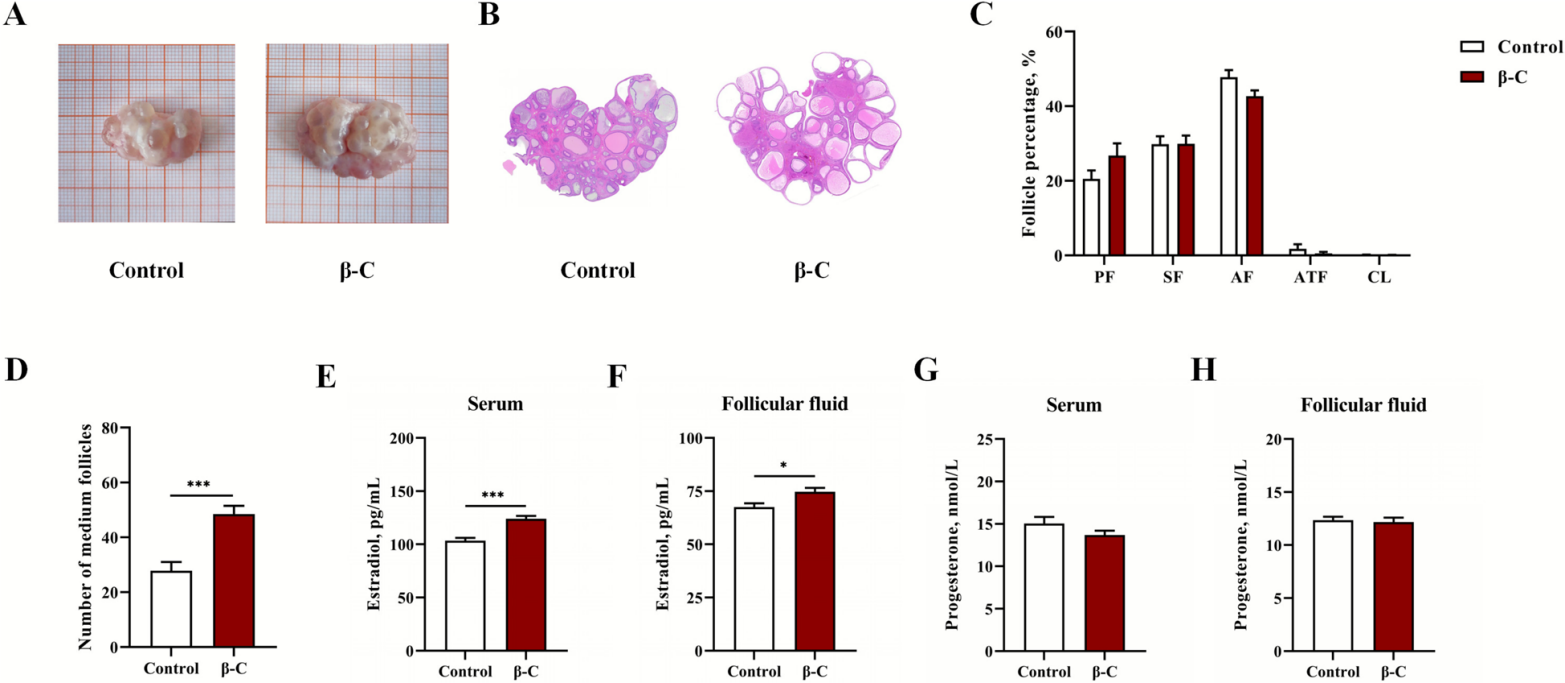
β-Carotene promotes follicular development of replacement gilts. **A** Gross morphology of ovaries. **B** Hematoxylin and Eosin (H&E) stained micrographs of ovarian tissue sections. **C** Proportions of ovarian follicles at various developmental stages. **D** Number of medium follicles. Concentrations of estradiol in serum (**E**) and follicular fluid (**F**). Concentrations of progesterone in serum (**G**) and follicular fluid (**H**). Data were shown as mean ± SEM, *n* = 10 replicates per treatment, representing the full cohort. ^*^P < 0.05, ^*^^*^P < 0.01, ^*^^*^^*^P < 0.001. PF: primary follicle; SF: secondary follicle; AF: antral follicle; ATF: atretic follicle; CL: corpus luteum

The weights of the ovaries, uterus, and oviducts, as well as the lengths of the uterine horns and oviducts, were measured. No significant differences in these reproductive organ parameters were observed between the control and β-C groups (*P* > 0.05, Table 2). To assess follicular development, we first quantified the number of large (> 5 mm), medium (2–5 mm), and small (< 2 mm) follicles on the ovarian surface. Subsequently, the number of follicles at each developmental stage was assessed histologically in consecutive sections (Fig. 1B). The proportions of primary, secondary, antral, and atretic follicles, as well as the corpus luteum, did not differ significantly between the two groups (*P* > 0.05, Fig. 1C). However, the β-C group exhibited a significantly higher number of medium follicles (2 mm ≤ diameter ≤ 5 mm) on the ovarian surface compared to the control group (*P* < 0.05, Fig. 1D). Furthermore, concentrations of estradiol in both serum and follicular fluid were significantly elevated in the β-C group (*P* < 0.05, Fig. 1E and F). In contrast, no significant differences were found in progesterone levels in either serum or follicular fluid between the groups (*P* > 0.05, Fig. 1G and H).

**Table 2:** Effects of dietary β-carotene supplementation on the development of reproductive tracts of gilts

Items	Control	β-C	*P*-value	SEM
Weight of ovaries, g	8.486	9.166	0.449	89.97
Weight of uterine, g	356.0	284.2	0.179	0.883
Length of left uterine horn, cm	63.79	56.17	0.224	6.117
Length of right uterine horn, cm	61.21	57.49	0.550	6.155
Weight of left oviduct, g	1.751	1.519	0.377	0.258
Weight of right oviduct, g	1.776	1.709	0.805	1.352
Length of left oviduct, cm	19.84^a^	16.21^b^	0.013	0.271
Length of right oviduct, cm	18.72	18.20	0.758	1.657

Within a row, means with different letters are significantly different (*P* < 0.05). *n* = 10 replicates per treatment, representing the full cohort

### β-Carotene accumulation in follicular fluid and back fat following dietary supplementation

**Fig. 2:**
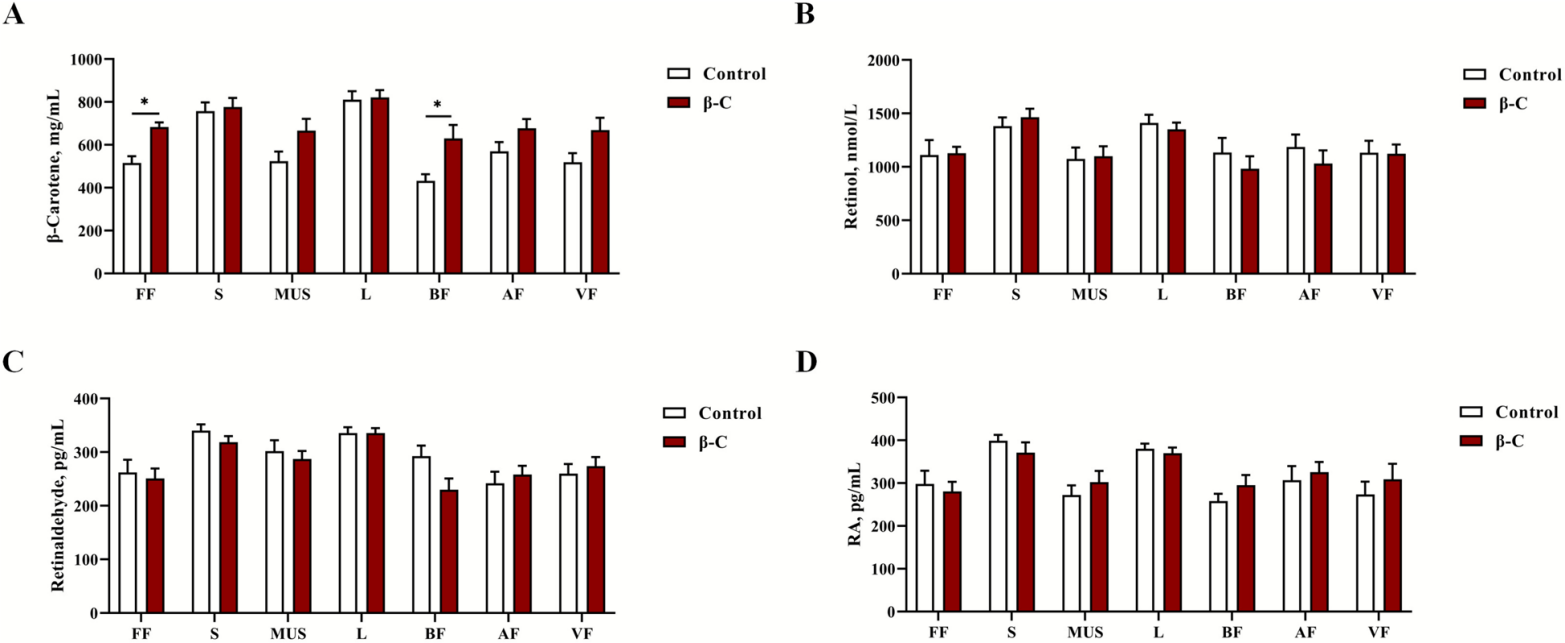
Concentrations of β-carotene and its metabolites in various tissues and organs. The concentrations of β-carotene (**A**), retinol (**B**), retinaldehyde (**C**) and retinoic acid (**D**) in various tissues and organs. Data were shown as mean ± SEM, *n* = 8 replicates per treatment. ^*^P < 0.05, ^*^^*^P < 0.01, ^***^P < 0.001. FF: follicle fluid; S: serum; MUS: muscle; L: liver; BF: back fat; AF: abdominal fat; VF: visceral fat

Concentrations of β-carotene and its metabolites (retinol, retinaldehyde, and RA) were measured in follicular fluid, serum, muscle, liver, back fat, abdominal fat, and visceral fat (Fig. 2). Dietary β-carotene supplementation significantly increased β-carotene levels specifically in follicular fluid and back fat (*P* < 0.05; Fig. 2A). In contrast, no significant differences were observed in the concentrations of retinol, retinaldehyde, or RA between the β-carotene and control groups across all tissues examined (*P* > 0.05; Fig. 2B–D).

### Identification of *FOXL2* as a differentially expressed gene in response to β-carotene

**Fig. 3:**
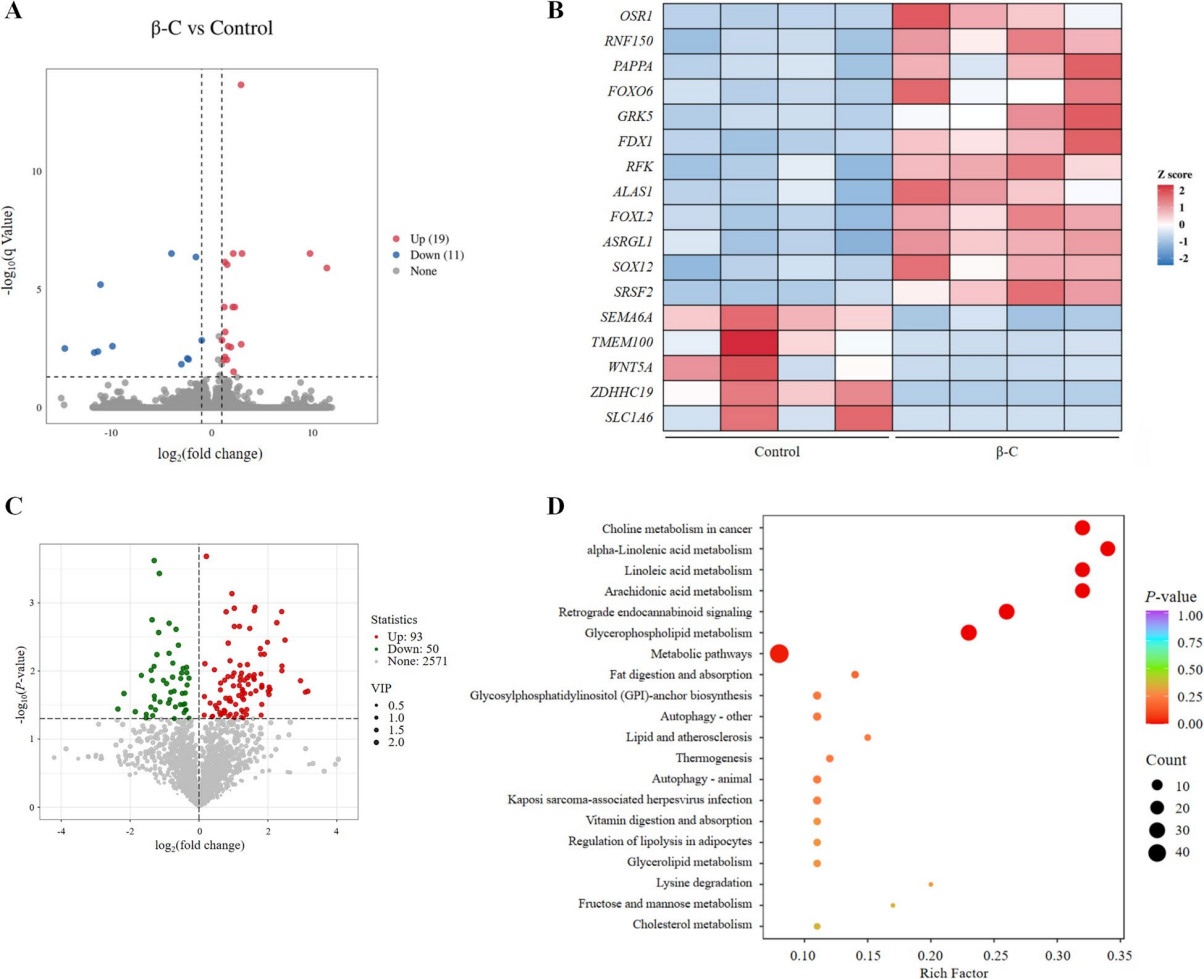
Differential analyses of GCs transcriptomic and follicular fluid metabolomic. **A** Volcano plot displays upregulated (red) and downregulated (blue) genes. **B** Heat map represents the expression levels of DEGs. **C** Volcano plot showcases upregulated (red) and downregulated (green) metabolites. **D** Enrichment analysis of differential metabolites by KEGG pathway. *n* = 4 replicates per treatment. A homogeneous subset of replacement gilts in proestrus was selected for omics analyses to minimize cycle-related variation

Transcriptomic sequencing of ovarian GCs revealed 19 upregulated and 11 downregulated genes in the β-C group compared to the control group (Fig. 3A). Among these, *FOXL2* was identified as a significantly upregulated differentially expressed gene (*P* < 0.05; Fig. 3B). Parallel metabolomic analysis of follicular fluid showed that 93 metabolites were significantly increased and 50 were decreased in the β-C group (Fig. 3C). These differential metabolites included a notable number of lipids and lipid-like molecules, such as lysophosphatidylcholines (LPCs) and fatty acid amides. These differential metabolites were enriched in pathways related to glycerophospholipid metabolism, linoleic acid metabolism, alpha-linolenic acid metabolism, and arachidonic acid metabolism (Fig. 3D).

**Fig. 4:**
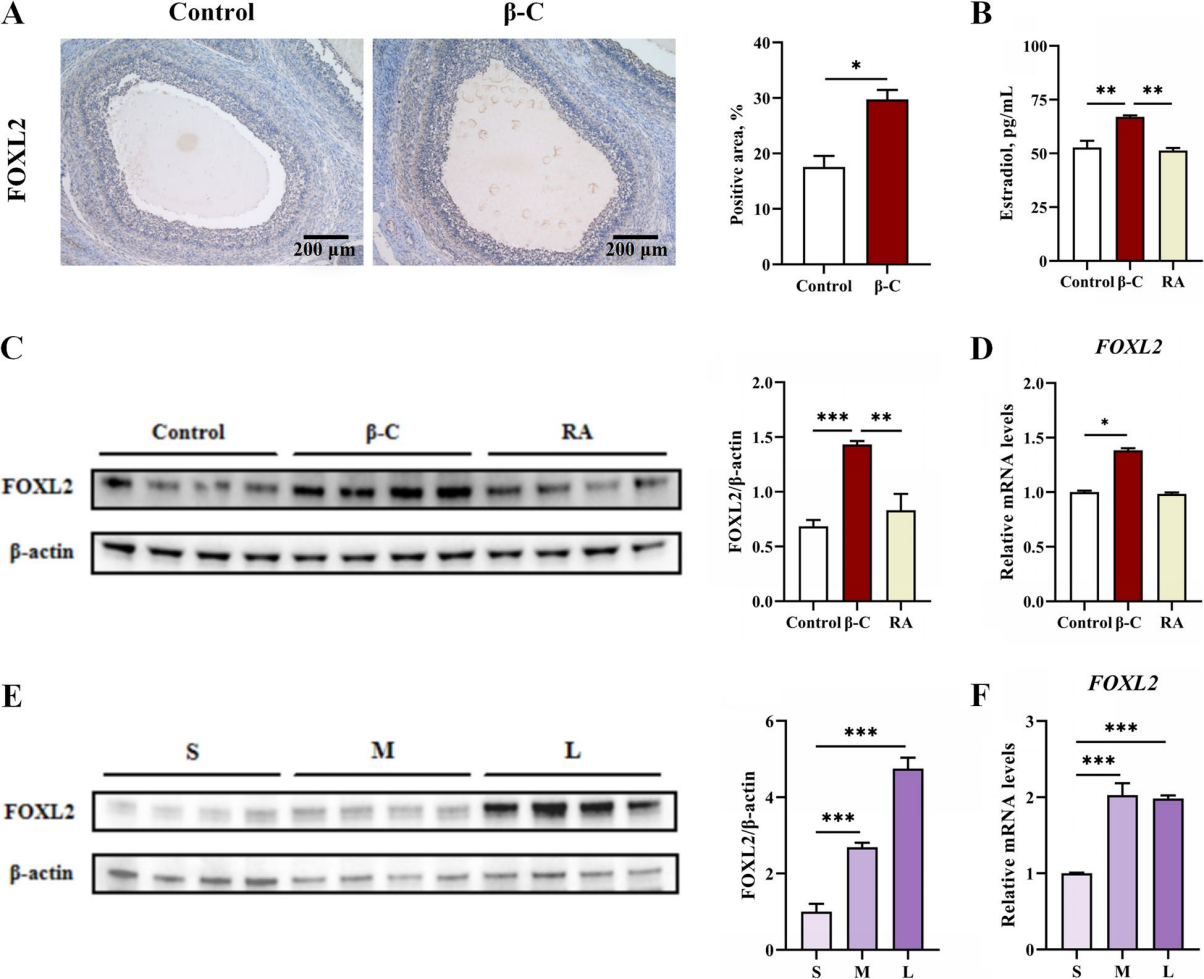
FOXL2 is an important target for β-carotene to promote ovarian follicle development. **A** Immunohistochemical staining results of FOXL2 in ovary. Scale bars, 200 μm. *n* = 6. Select ovarian specimens with the largest cross-section capable of being fully displayed on a microscope slide for statistical analysis. **B** The estradiol concentration in GCs of control group, β-C group and RA group. *n* = 4. Relative protein levels (**C**) and mRNA levels (**D**) of FOXL2 in GCs of control group, β-C group and RA group. *n* = 4. Relative protein levels (**E**) and mRNA levels (**F**) of FOXL2 in GCs from follicles of different sizes. *n* = 4. Data were shown as mean ± SEM. ^*^P < 0.05, ^**^P < 0.01, ^*^^*^^*^P < 0.001. S: small follicle; M: medium follicle; L: large follicle

To validate the transcriptomic findings, immunohistochemical staining was performed on ovarian sections. The positive signal proportion for FOXL2 was significantly higher in the β-C group than in the control group (*P* < 0.05; Fig. 4A). Subsequently, in vitro experiments using GCs were conducted. Treatment with 2.5 μmol/L β-carotene had no adverse effect on GC viability (Fig. S1A) but significantly increased estradiol concentrations in the culture medium (Fig. S1B). Based on this, 2.5 μmol/L β-carotene was selected for subsequent in vitro treatments.

Given that RA is an active VA metabolite[22], its effects were compared to those of β-carotene. After 24-h treatment, the β-C group, but not the RA group, exhibited significantly elevated FOXL2 expression in GCs and increased estradiol levels in the medium compared to the control (*P* < 0.05; Fig. 4B−D). Furthermore, analysis of GCs isolated from follicles of different sizes showed that FOXL2 expression at both the gene and protein levels increased significantly with follicular maturation (*P* < 0.05; Fig. 4E and F).

### Roles of FOXL2 in regulating steroidogenic genes and estradiol synthesis

**Fig. 5:**
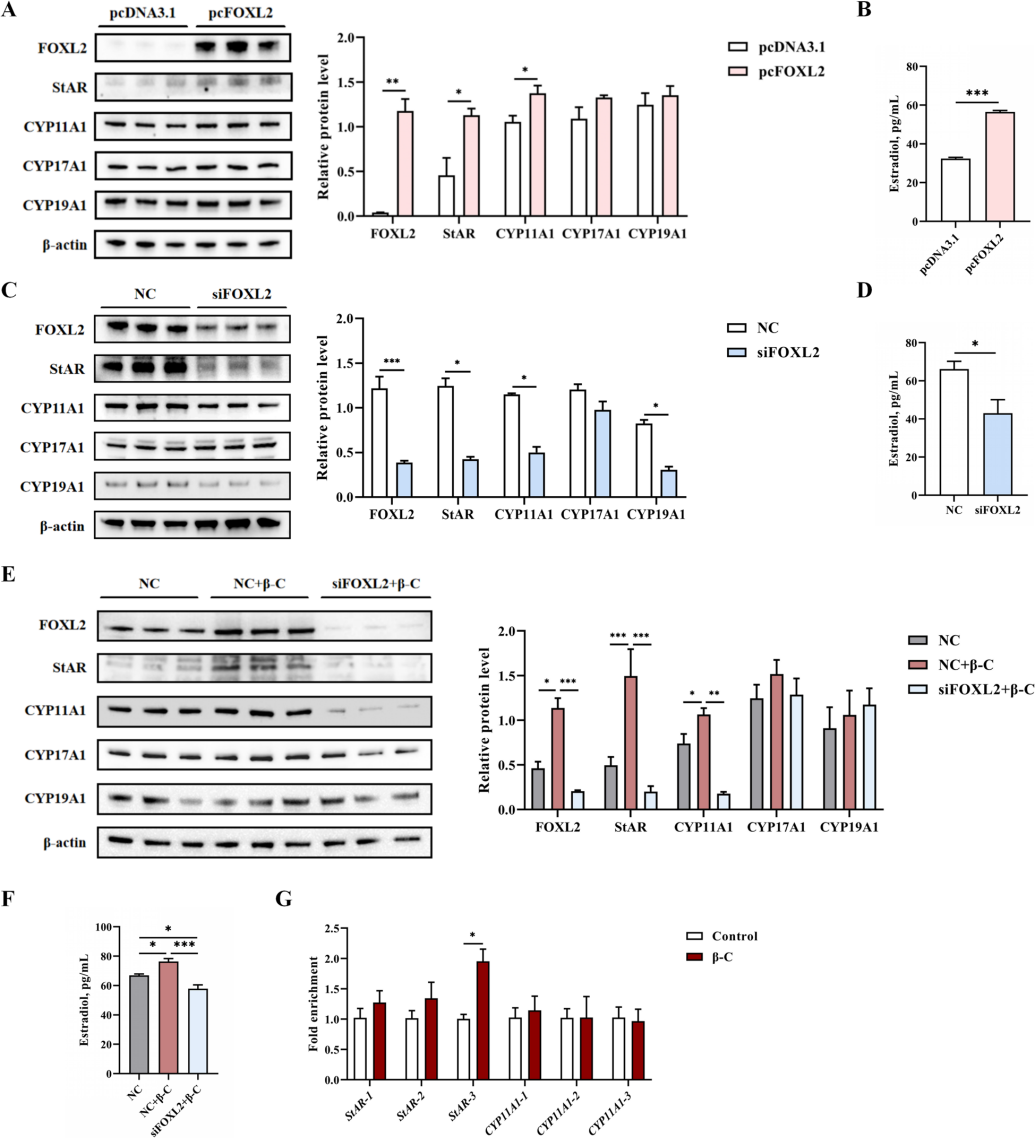
β-Carotene mediates FOXL2 expression, regulates StAR, and affects estradiol synthesis in GCs. The relative protein levels of genes related to estradiol synthesis in GCs were assessed following the overexpression (**A**) or interference (**C**) of FOXL2. Estradiol concentrations of GCs culture medium were measured after overexpression (**B**) or interference (**D**) with FOXL2. **E** The effect of β-carotene to elevate the expression of FOXL2 and estradiol synthesis related proteins disappeared when supplemented with β-carotene for 24 h after interfering with FOXL2 expression. **F** The effect of β-carotene to elevate estradiol synthesis disappeared when supplemented with β-carotene for 24 h after interfering with FOXL2 expression. **G** Interaction of FOXL2 with *StAR* and *CYP11A1*. Data were shown as mean ± SEM, *n* = 3 replicates per treatment. ^*^P < 0.05, ^**^P < 0.01, ^***^P < 0.001. StAR: steroidogenic acute regulatory protein; CYP11A1: cytochrome P450 family 11 subfamily A member 1; CYP17A1: cytochrome P450 family 17 subfamily A member 1; CYP19A1: cytochrome P450 family 19 subfamily A member

To investigate the function of FOXL2 in granulosa cells (GCs), gain- and loss-of-function experiments were performed. Overexpression of FOXL2 significantly increased the protein levels of StAR and CYP11A1, along with the concentration of estradiol in the culture medium, compared to the control group (*P* < 0.05; Fig. 5A and B). Conversely, FOXL2 interference significantly decreased the expression of StAR and CYP11A1, as well as estradiol levels (*P* < 0.05; Fig. 5C and D). Furthermore, when β-carotene was supplemented following FOXL2 interference, it failed to upregulate the expression of FOXL2, StAR, and CYP11A1, and did not increase estradiol levels (*P* < 0.05; Fig. 5E and F).

To explore the mechanism by which *FOXL2* regulates *StAR*, CUT&Tag experiments were conducted. The promoter sequences of *StAR* and *CYP11A1* were retrieved, and potential *FOXL2 *binding sites were predicted (Table S4). CUT&Tag-qPCR results demonstrated a significant enrichment of *FOXL2* binding specifically in the *StAR* promoter region after β-carotene treatment (*P* < 0.05; Fig. 5G). No significant enrichment was observed at the predicted binding sites on the *CYP11A1* promoter.

### Effects of β-carotene on lipid droplet content and expression of lipid metabolism-related genes

**Fig. 6:**
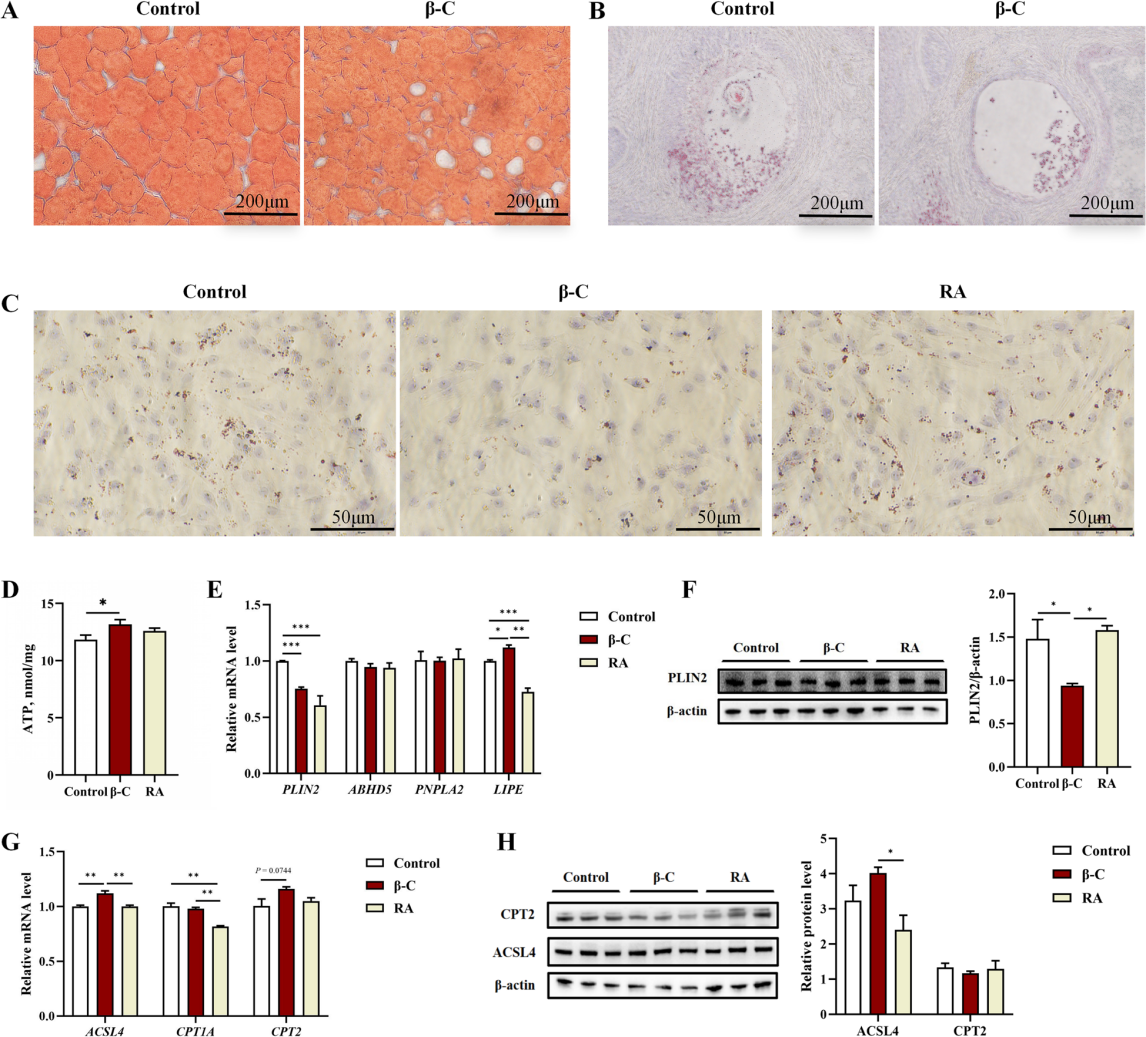
β-Carotene promotes lipolysis. **A**–**C** Representative images of Oil Red O staining in (**A**) back fat, (**B**) ovary, and (**C**) granulosa cells (GCs). Scale bar, 50 μm. (**A**, **B**) *n* = 5; (**C**) *n* = 3. **D** ATP production in GCs. *n* = 6. **E** Relative mRNA expression levels of lipolysis-related genes (*PLIN2*, *ABHD5*, *PNPLA2*, *LIPE*) in GCs from control, β-carotene, and retinoic acid (RA) groups. *n* = 3. **F** Representative Western blot (left) and quantitative analysis (right) of PLIN2 protein expression in GCs. Protein levels were normalized to β-actin. *n* = 5. **G** Relative mRNA expression levels of genes involved in fatty acid activation and oxidation in GCs. *n* = 3. **H** Representative Western blots (left) and quantitative analysis (right) of ACSL4 and CPT2 protein expression in GCs. Protein levels were normalized to β-actin. *n* = 5. Data are presented as mean ± SEM. Statistical significance is indicated as ^*^P < 0.05, ^**^P < 0.01, ^***^P < 0.001. ATP: adenosine triphosphate; PLIN2: perilipin 2; ABHD5: abhydrolase domain containing 5; PNPLA2: patatin-like phospholipase domain containing 2; LIPE: lipase, hormone-sensitive; ACSL4: acyl-CoA synthetase long-chain family member 4; CPT1A: carnitine palmitoyltransferase 1 A; CPT2: carnitine palmitoyltransferase 2

Compared with the control group, the β-C group exhibited a significant reduction in lipid droplets in both dorsal fat tissue and ovarian follicles (*P* < 0.05; Fig. 6A and B). In vitro, granulosa cells (GCs) treated with β-carotene also showed significantly fewer lipid droplets and higher ATP levels compared to both the control and RA groups (*P* < 0.05; Fig. 6C and D).

The expression of key genes involved in lipid metabolism was examined in GCs. Regarding lipid droplet stability and lipolysis, β-carotene treatment significantly downregulated the mRNA and protein levels of PLIN2 compared to the control group (*P* < 0.05; Fig. 6E and F). In contrast, RA treatment downregulated PLIN2 mRNA but did not affect its protein level (Fig. 6E and F). For core lipolytic enzymes, β-carotene treatment significantly upregulated the mRNA expression of *LIPE*, whereas RA treatment markedly suppressed it (*P* < 0.05; Fig. 6E). No significant differences were observed in the mRNA expression levels of *ABHD5* and *PNPLA2* among the groups (*P* > 0.05; Fig. 6E).

Regarding the fatty acid oxidation pathway, β-carotene treatment significantly upregulated the mRNA and protein levels of ACSL4 (*P* < 0.05; Fig. 6G and H). RA treatment did not alter ACSL4 expression (*P* > 0.05; Fig. 6G and H). For *CPT1A*, mRNA levels in both the β-C and control groups were significantly higher than in the RA group (*P* < 0.05; Fig. 6G). For *CPT2*, the β-C group showed a non-significant trend towards increased mRNA levels compared to the control group (*P* = 0.074; Fig. 6G), and no significant differences in CPT2 protein levels were detected (*P* > 0.05; Fig. 6H).

### β-Carotene reduces ROS levels and increases antioxidant enzyme expression and activity

**Fig. 7:**
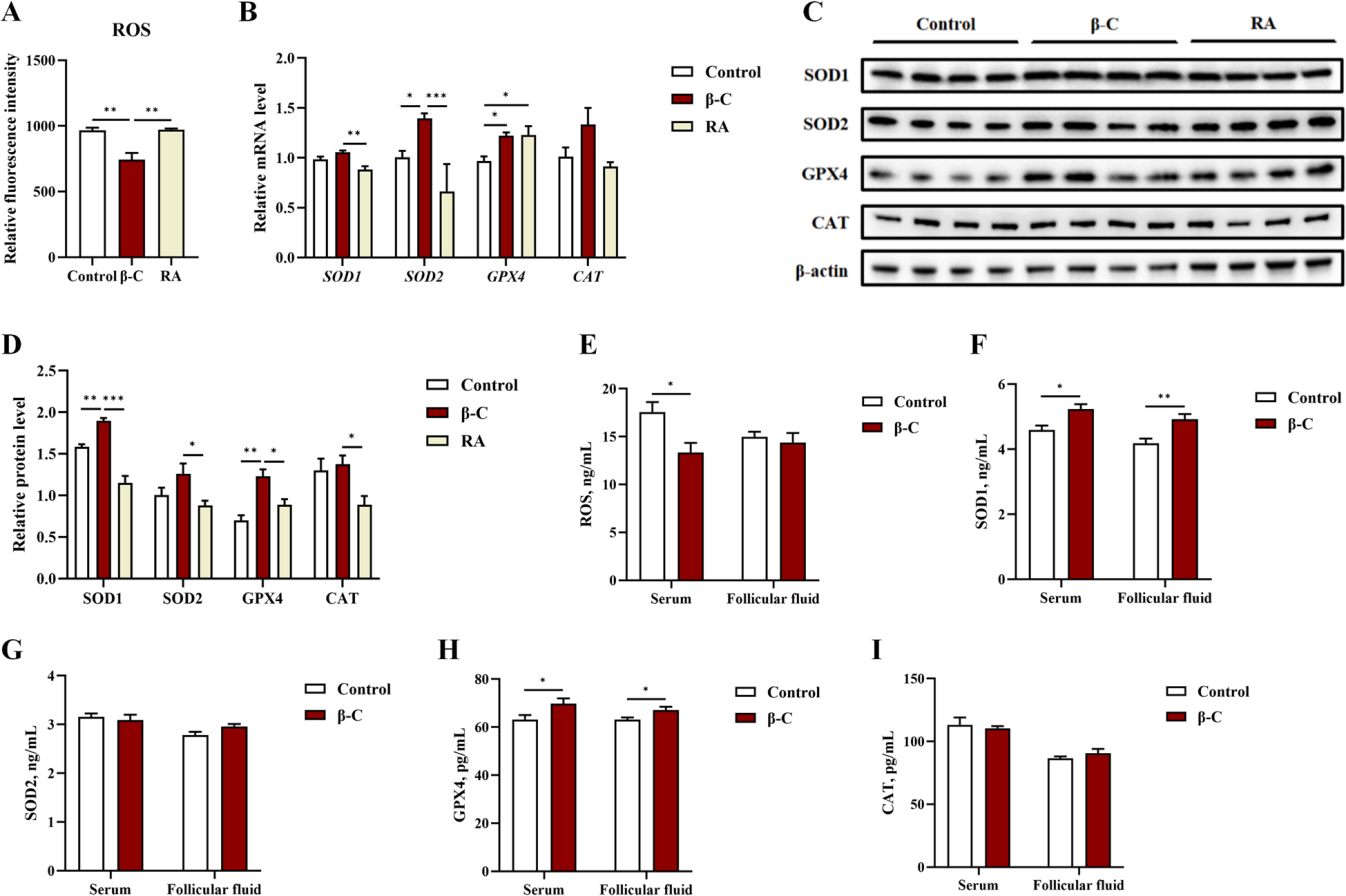
β-Carotene reduced oxidative stress. **A** ROS levels of GCs (*n* = 4). Relative mRNA levels (**B**) and protein levels (**C** and **D**) of antioxidant enzyme in GCs following treatment with β-carotene or RA for 24 h (*n* = 3). ROS concentration (**E**) and antioxidant enzyme activity (**F**–**I**) in serum and follicular fluid of replacement gilts (*n* = 5). The sample size reflects the maximum number of animals with complete high-quality data for all assays compared in this analysis. Data were shown as mean ± SEM. ^*^P < 0.05, ^**^P < 0.01, ^***^P < 0.001. ROS: reactive oxygen species; SOD1: superoxide dismutase 1; SOD2: superoxide dismutase 2; GPX4: glutathione peroxidase 4; CAT: catalase

In granulosa cells (GCs), ATP production was significantly higher in the β-C group compared to the control and RA groups, while intracellular ROS levels were significantly lower (*P* < 0.05; Fig. 7A). The expression levels of the antioxidant enzymes SOD1 and GPX4 were also significantly increased in GCs from the β-C group (*P* < 0.05; Fig. 7B–D).

Consistent with the in vitro findings, the in vivo analysis showed that dietary β-carotene supplementation significantly decreased serum ROS levels in gilts (*P* < 0.05; Fig. 7E). Furthermore, the enzyme activity of SOD1 and GPX4 was significantly increased in both the serum and follicular fluid of the β-C group (*P* < 0.05; Fig. 7F–I). In contrast, no significant changes were observed in the activities of catalase (CAT) or superoxide dismutase 2 (SOD2; *P* > 0.05).

## Discussion

The ovary is an organ that undergoes dynamic changes at the morphological, intracellular cytological, molecular, protein, and signaling pathway levels [23, 24]. Recent studies have focused on the regulation of GCs function [25, 26], oocyte quality [27], and follicle development [28] through the exogenous supplementation of nutrients. In this study, the supplementation of β-carotene in replacement gilts resulted in a significant increase in the number of medium follicles and estradiol levels in follicular fluid, indicating that β-carotene positively influences follicular development and the initiation of estrus in replacement gilts. Previous research has reported that the metabolite of β-carotene, RA, can regulate GC proliferation and estradiol synthesis [21]. In this study, following the dietary addition of β-carotene, the concentration of β-carotene in the follicular fluid of replacement gilts significantly increased, while the levels of its metabolites, retinol, retinaldehyde, and RA, remained unchanged. Based on these findings, we propose a hypothesis that the increase in the number of medium follicles and estradiol levels in follicular fluid in gilts supplemented with β-carotene in this experiment is attributable to the action of β-carotene itself, rather than to its metabolites.

We annotated *FOXL2* among the differentially expressed genes of GCs in the comparison between the control group and β-C group. FOXL2 is closely associated with the differentiation and maintenance of GCs and serves as a crucial transcription factor for sustaining ovarian phenotype and function [29–31]. During gonadal development and follicle formation, FOXL2 interacts with various partners to ensure normal ovarian development and function, including ubiquitin-specific protease 7 (USP7) [32]. Deficiency or mutation of FOXL2 can lead to female reproductive disorders. In adult ovaries, granulosa and theca cells can transdifferentiate into long-lived Sertoli or Leydig cells following FOXL2 deficiency [31]. A recurrent mutation in FOXL2 (402C → G) is commonly observed in adult-type GC tumors [33]. Additionally, loss-of-function mutations in FOXL2 are associated with blepharophimosis ptosis-epicanthus inversus syndrome in females with primary ovarian insufficiency [34]. Based on these established roles of FOXL2 in ovarian function, we hypothesize that FOXL2 is a key target for β-carotene in promoting follicle development and steroid hormone synthesis.

To elucidate the role of FOXL2, we conducted FOXL2 interference and overexpression treatments, revealing that estradiol levels and estradiol synthesis-related proteins, specifically StAR and CYP11A1, exhibited consistent trends in relation to FOXL2 expression. StAR governs the initial critical event in estradiol synthesis by mediating the transport of cholesterol into mitochondria [35]. CYP11A1, located on the inner mitochondrial membrane, catalyzes the side-chain cleavage reaction of cholesterol, which represents the first and rate-limiting step in estradiol synthesis [36]. Our CUT&Tag data provide direct evidence that FOXL2 binds to the promoter-associated region of StAR, suggesting a mechanism for the enhanced estradiol synthesis. The coordinated upregulation of CYP11A1 expression with FOXL2 suggests its potential involvement in this regulatory network; however, a direct transcriptional mechanism for CYP11A1 was not established in our study. This finding aligns with previous studies demonstrating that FOXL2 functions as a transcription factor that binds to StAR, and that FOXL2 plays a more significant role during the sexual maturation stage in mice compared to the embryonic stage [32, 37].

Analysis of follicular fluid metabolomics data has revealed that the differential metabolites between the β-C group and the control group are closely associated with polyunsaturated fatty acid metabolism and lipid metabolism. There are some studies indicating that β-carotene holds great potential for preventing and reducing obesity. As a bioactive molecule prevalent in various foods, β-carotene presents a safer alternative for obesity management compared to approved synthetic drugs [38]. Recent evidence indicates that β-carotene reduces lipid synthesis in 3T3-L1 cells, enhances fatty acid oxidation, stimulates beige fat gene and protein expression, and increases thermogenesis [39]. We therefore hypothesise that β-carotene alters lipid breakdown and fatty acid oxidation in gilts. The finding that β-carotene supplementation significantly increased β-carotene accumulation in dorsal adipose tissue and markedly reduced lipid droplets in ovarian sections corroborates our hypothesis. Subsequently, to test this hypothesis at the cellular level, we examined key molecules involved in lipid droplet stability, lipolysis, and fatty acid activation/oxidation within granulosa cells. Our results suggest that β-carotene modulates lipid metabolism through coordinated mechanisms: firstly, it may enhance lipolysis by reducing PLIN2 (a lipid droplet stabilising protein [40, 41]) and upregulating *LIPE* (the gene encoding a lipolysis rate-limiting enzyme [42, 43]); while simultaneously promoting free fatty acid activation through specific upregulation of ACSL4 (an enzyme committing activated fatty acids toward β-oxidation [44]). These findings support a novel role for β-carotene in regulating ovarian granulosa cell energy metabolism, independent of classical RA signalling pathways.

The increase in ATP production in GCs following β-carotene treatment, alongside a decrease in ROS levels, presents an intriguing paradox. β-Carotene, a prevalent antioxidant found in both dietary sources and nature, has been extensively studied in animal models in recent years. Notably, the incorporation of β-carotene into the diets of sows during late pregnancy significantly enhances GSH-Px activity [15]. This augmentation of antioxidant capacity appears to be transmitted from sows to piglets through the placenta and colostrum [18]. Based on our data, we propose a model to resolve this paradox: under the lipolytic influence of β-carotene, both ATP and ROS production may increase in GCs. The upregulation of intracellular SOD1 and GPX4 in GCs likely enhances ROS scavenging, supporting redox homeostasis. This adaptation aligns with reduced GC ROS levels and correlates with elevated antioxidant activities in follicular fluid. Following ovulation, replacement gilts exhibit higher levels of oxidative stress compared to sows [45]. Therefore, dietary addition of β-carotene in replacement gilts represents a promising nutritional strategy that mitigates oxidative stress and enhances productivity.

Our research has revealed the pivotal role of β-carotene in GCs on energy metabolism, estradiol production, redox balance, and the regulation of ovarian development. However, this study is subject to several limitations. The sample size was relatively small, and to account for natural variations in hormone levels, synchronized oestrus induction was not employed, thereby avoiding the ‘high background noise’ associated with such protocols. Nevertheless, this approach cannot entirely rule out the influence of individual variability or the differing stages of the oestrous cycle. Furthermore, concentrations of β-carotene and retinoids in various tissues and organs were measured using ELISA without confirmatory chromatographic analysis. Although clear intergroup trends and high intragroup consistency were observed, employing chromatographic technologies would provide greater specificity and precision. In addition, the concentrations of β-carotene and retinoids were measured only at the endpoint of the study. This single time-point measurement provides a static snapshot and does not capture the potential dynamic changes in their metabolic kinetics throughout the trial. Furthermore, mechanistic investigations of β-carotene were conducted using in vitro granulosa cells (GCs) derived from slaughterhouse ovaries rather than experimental gilts. However, the successful in vitro recreation of core phenotypes, namely, β-carotene-specific induction of increased oestradiol synthesis and reduced lipid droplet formation, supports the biological relevance of our in vitro findings for interpreting the in vivo observations. Moreover, the effect of β-carotene on the expression of genes such as *FOXL2*, *PLIN2*, and *ACSL4* warrants further investigation. It remains to be determined whether this effect is mediated through the direct interaction of β-carotene as a signaling molecule with transcription factors or nuclear receptors, or if it is a consequence of epigenetic modifications induced by β-carotene.

## Conclusion

Our research indicates that β-carotene contributes to ovarian development by coordinating estradiol synthesis, lipid metabolism, and redox homeostasis. Our data support a model in which β-carotene upregulates the FOXL2-StAR axis to enhance estradiol synthesis. Furthermore, β-carotene triggers a metabolic shift in granulosa cells, characterized by downregulation of PLIN2 and upregulation of LIPE and ACSL4, which is associated with increased ATP production. This energetic shift occurred alongside an upregulation of intracellular antioxidant enzymes (SOD1, GPX4) and a net reduction in cellular ROS levels, indicating an enhanced capacity to maintain redox balance under metabolic activation. Our findings not only provide a theoretical basis for β-carotene application in gilts but also offer a scientific rationale for its use as a nutritional intervention to support ovarian development and oestrus initiation.

## Supplementary Information

Additional file 1: Table S1. List of primers used for Real-time PCR. Table S2. List of primary antibodies used for Western blot analysis. Table S3. siRNA sequences. Table S4. List of primers used for CUT&Tag-PCR.

Additional file 2: Fig. S1. Dose-dependent effects of β-carotene and its metabolites on granulosa cell viability and estradiol secretion.

Additional file 3. The full Western blot images.

Additional file 4. List of differential metabolites between β-C and control groups.

## Data Availability

The data that support the findings of this study are available from the corresponding author upon reasonable request. The transcriptome data generated in this study have been deposited in the NCBI Sequence Read Archive (SRA) under the BioProject accession number PRJNA1371885. The metabolomics data have been deposited in the MetaboLights database under accession code MTBLS13556.

## Abbreviations

ATP: Adenosine triphosphate
ABHD5: Abhydrolase domain containing 5
ACSL4: Acyl-CoA synthetase long-chain family member 4
β-C: β-Carotene
COCs: Cumulus-oocyte complexes
CPT1A: Carnitine palmitoyltransferase 1A
CPT2: Carnitine palmitoyltransferase 2
CAT: Catalase
CYP11A1: Cytochrome P450 family 11 subfamily A member 1
FOXL2: Forkhead box L2
FFAs: Free fatty acids
GSH-Px: Glutathione peroxidase
GPX4: Glutathione peroxidase 4
GCs: Granulosa cells
IL-4: Interleukin 4
LPCs: Lysophosphatidylcholines
LIPE: Hormone-sensitive lipase
PNPLA2: Patatin-like phospholipase domain containing 2
PLIN2: Perilipin 2
ROS: Reactive oxygen species
RA: Retinoic acid
StAR: Steroidogenic acute regulatory protein
SOD1: Superoxide dismutase 1
SOD2: Superoxide dismutase 2
TNF-α: Tumor necrosis factor α
USP7: Ubiquitin-specific protease 7
VA: Vitamin A
