# Supplementary material for: Dietary β-carotene improves the ovary development and antioxidant capacity of replacement gilts
Source: J Anim Sci Biotechnol. 2026 Feb 7;17:23. doi: 10.1186/s40104-025-01342-2 (PMC12882461; doi:10.1186/s40104-025-01342-2)
Supplement: Supplementary file 1 — Additional file 1: Table S1. List of primers used for Real-time PCR. Table S2. List of primary antibodies used for Western blot analysis. Table S3. siRNA sequences. Table S4. List of primers used for CUT&Tag-PCR. [file 40104_2025_1342_MOESM1_ESM.docx]

**Table S1.** List of primers used for Real-time PCR.

| **Targeting gene name** | **Primer sequence （5’ to 3’）** | **GenBank Accession No.** | **Amplicon Length (bp)** | **Final Concentration (μM)** |
| --- | --- | --- | --- | --- |
| *Sus scrofa* |  |  |  |  |
| β-Actin | F: TGCGGGACATCAAGGAGAAGC | XM_021086047.1 | 273 | 10 |
|  | R: ACAGCACCGTGTTGGCGTAGAG |  |  |  |
| FOXL2 | F: GAGAAGAGGCTCACGCTGTCCG | NM_001244665.1 | 111 | 10 |
|  | R: GAGGCTGAGGTTGTGGCGAAT |  |  |  |
| PLIN2 | F: TGTGAGATGGCAGAGAAGGG | NM_214200.2 | 198 | 10 |
|  | R: CACAGCCCCTTTAGCATTGG |  |  |  |
| ABHD5 | F: ATGCTCCATCGGATTGGTAA | NM_001012407.1 | 153 | 10 |
|  | R: ATGTCCTGCTCCAAGAATGG |  |  |  |
| PNPLA2 | F: GACGGTGGCATCTCAGACAA | NM_001098605.1 | 113 | 10 |
|  | R: TGGATGTTGGTGGAGCTGTC |  |  |  |
| LIPE | F: GCCTTTCCTGCAGACCATCT | NM_214315.3 | 104 | 10 |
|  | R: CACTGGTGAAGAGGGAGCTG |  |  |  |
| ACSL4 | F: GATTGACAGAATCGTGTGGCG | NM_001038694.1 | 187 | 10 |
|  | R: CCCATGGAGATATTCTGTCCACC |  |  |  |
| CPT1A | F: GCATTTGTCCCATCTTTCGT | NM_001129805.2 | 199 | 10 |
|  | R: GCACTGGTCCTTCTGGGATA |  |  |  |
| CPT2 | F: CAAGGCCTACCCTCTGGATA | NM_001246243.1 | 175 | 10 |
|  | R: GCTCACAATCTTCCCGTCTT |  |  |  |
| SOD1 | F: ATCAAGAGAGGCACGTTGGA | NM_001190422.1 | 158 | 10 |
|  | R: TCTGCCCAAGTCATCTGGTT |  |  |  |
| SOD2 | F: TCAAGGAGAAGTTGACCGCT | NM_214127.2 | 181 | 10 |
|  | R: AGGTAATACGCATGCTCCCA |  |  |  |
| GPX4 | F: GATTCTGGCCTTCCCTTGC | NM_214407.1 | 173 | 10 |
|  | R: TCCCCTTGGGCTGGACTTT |  |  |  |
| CAT | F: AGATGGACACAGGCACATGA | NM_214301.2 | 172 | 10 |
|  | R: CCGGATGCCATAGTCAGGAT |  |  |  |

β-Actin = beta-actin; FOXL2 = forkhead box L2; PLIN2 = perilipin 2; ABHD5 = abhydrolase domain containing 5; PNPLA2 = patatin-like phospholipase domain containing 2; LIPE = hormone-sensitive lipase; ACSL4 = acyl-CoA synthetase long-chain family member 4; CPT1A = carnitine palmitoyltransferase 1A; CPT2 = carnitine palmitoyltransferase 2; SOD1 = superoxide dismutase 1; SOD2 = superoxide dismutase 2; GPX4 = glutathione peroxidase 4; CAT = catalase.

**Table S2.** List of primary antibodies used for western blot analysis.

| Antibody | Molecular weight | Source | Catalog Code |
| --- | --- | --- | --- |
| FOXL2 | 39-50 kDa | Proteintech | 19672-1-AP |
| FOXL2 | 39-50 kDa | Abways | CY8500 |
| STAR | 32 kDa | Proteintech | Cat No. 12225-1-AP |
| CYP11A1 | 60 kDa | Abways | AY1858 |
| CYP17A1 | 50-57 kDa | Abways | CY8577 |
| CYP19A1 | 53-58 kDa | Abways | CY6731 |
| PLIN2 | 48-55 kDa | Abways | CY6811 |
| ACSL4 | 79 kDa | Abways | DY1198 |
| CPT2 | 74 kDa | Abways | CY5699 |
| SOD1 | 16-18kDa | Abways | CY6666 |
| SOD2 | 23kDa | Abways | CY5977 |
| GPX4 | 17-22kDa | Abways | CY6959 |
| CAT | 60kDa | Abways | CY6783 |
| β-ACTIN | 42 kDa | Proteintech | Cat No. 66009-1-Ig |
| β-ACTIN | 42 kDa | Proteintech | Cat No. 20536-1-AP |

FOXL2 = forkhead box L2 protein; STAR = steroidogenic acute regulatory protein; CYP11A1 = cytochrome P450 family 11 subfamily A member 1; CYP17A1 = cytochrome P450 family 17 subfamily A member 1; CYP19A1 = cytochrome P450 family 19 subfamily A member; PLIN2 = perilipin 2; ACSL4 = acyl-CoA synthetase long-chain family member 4; CPT2 = carnitine palmitoyltransferase 2; SOD1 = superoxide dismutase 1; SOD2 = superoxide dismutase 2; GPX4 = glutathione peroxidase 4; CAT = catalase; β-ACTIN = beta-actin.

**Table S3.** siRNA sequences.

|  | Forwards (5’ to 3’) | Reverses (5’ to 3’) |
| --- | --- | --- |
| NC | UCCUCCGAACGUGUCACGUTT | ACGUGACACGUUCGGAGAATT |
| FOXL2 siRNA | CCGGCAUCUACCAGUAUAUTT | AUAUACUGGUAGAUGCCGGTT |

siRNA = small interfering RNA; NC = negative control; FOXL2 = forkhead box L2.

**Table S4.** List of primers used for CUT&Tag-PCR.

|  | Forwards (5’ to 3’) | Reverses (5’ to 3’) |
| --- | --- | --- |
| StAR-1 | CCTGGAGAGTATTATGCCAAATGA | ACCCTGAAAATGGCCAAAATGG |
| StAR-2 | AGTTTCCCATTTCAGCTCCCA | TGTCATTTCTCAAGTGGGCT |
| StAR-3 | AGAGTCCCAGCACTCCCAA | TGTGACAGTGGTTCTCAAGCA |
| CYP11A1-1 | CACTGAACTATTGCTTAAGCTCTCA | GAGCAGGCACAAGAAGTTTACA |
| CYP11A1-2 | TGTTTCTGCATTCCCTCCCT | GACAAGACCCCTCCATGTCC |
| CYP11A1-3 | ACAAGAATTAAGAACTAAGTGGGCT | CTTGCACCCCATTTTCCTGC |

CUT&Tag = Cleavage Under Targets and Tagmentation; STAR = steroidogenic acute regulatory protein; CYP11A1 = cytochrome P450 family 11 subfamily A member 1.
