# Supplementary material for: Dietary β-carotene improves the ovary development and antioxidant capacity of replacement gilts
Source: J Anim Sci Biotechnol. 2026 Feb 7;17:23. doi: 10.1186/s40104-025-01342-2 (PMC12882461; doi:10.1186/s40104-025-01342-2)
Supplement: Supplementary file 2 — Additional file 2: Fig. S1. Dose-dependent effects of β-carotene and its metabolites on granulosa cell viability and estradiol secretion. [file 40104_2025_1342_MOESM2_ESM.docx]

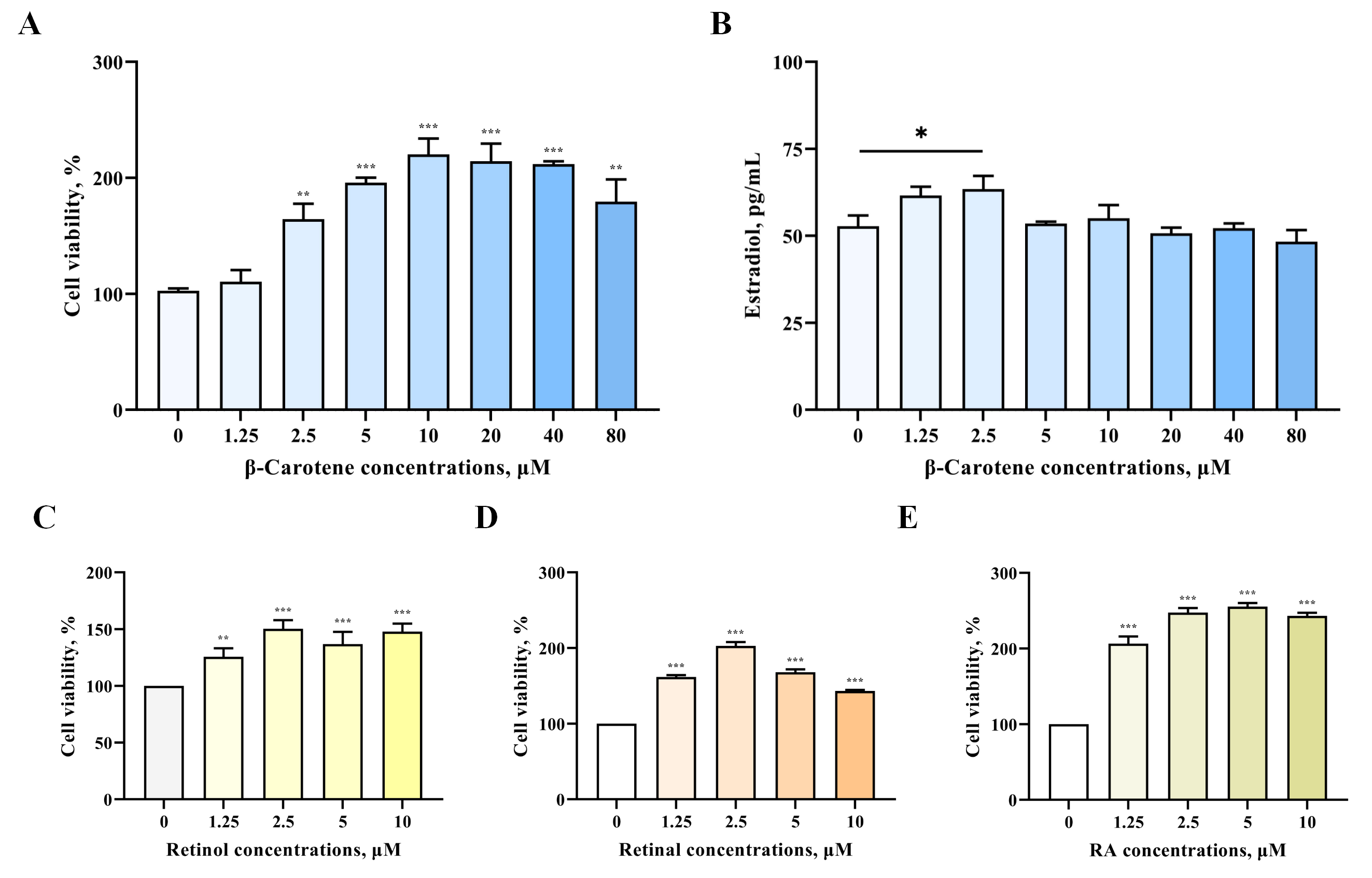


**Fig. S1.** Dose-dependent effects of β-carotene and its metabolites on granulosa cell viability and estradiol secretion. (A) The Cell viability of GCs treated with different doses of β-carotene. (B) The concentrations of estradiol of GCs treated with different doses of β-carotene. The Cell viability of GCs treated with different doses of retinol (C), retinal (D) and RA (E). Data were shown as mean ± SEM, *n* = 3 replicates per treatment. *P*-value less than 0.05 indicates a significant difference. *P* <0.05 *, *P* <0.01 **, *P* <0.001 ***. RA = retinoic acid.
