# Supplementary material for: Dietary β-carotene improves the ovary development and antioxidant capacity of replacement gilts
Source: J Anim Sci Biotechnol. 2026 Feb 7;17:23. doi: 10.1186/s40104-025-01342-2 (PMC12882461; doi:10.1186/s40104-025-01342-2)

**Figure 4C**

**FOXL2**

**
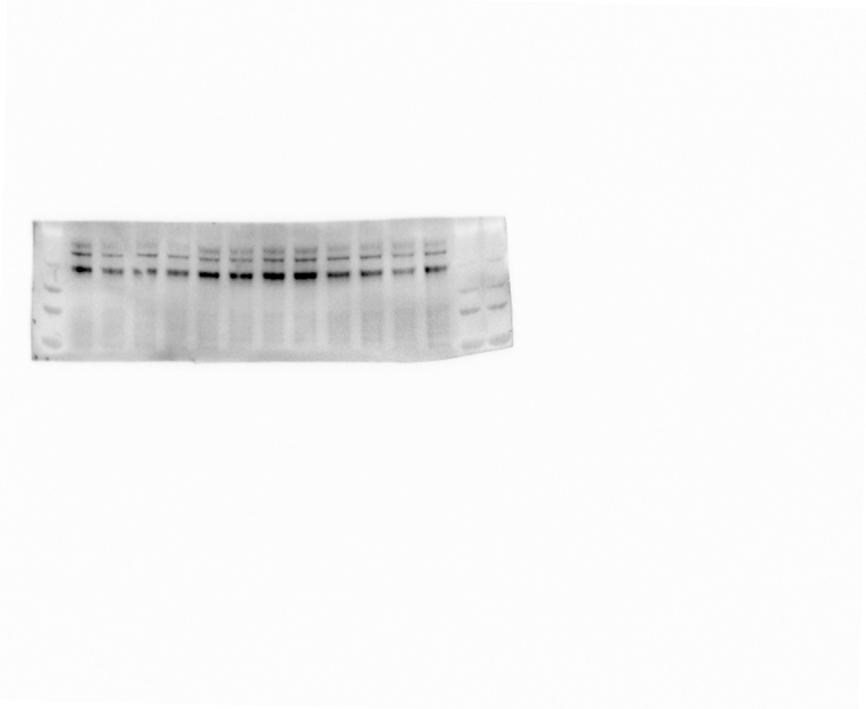
**

**β-actin**


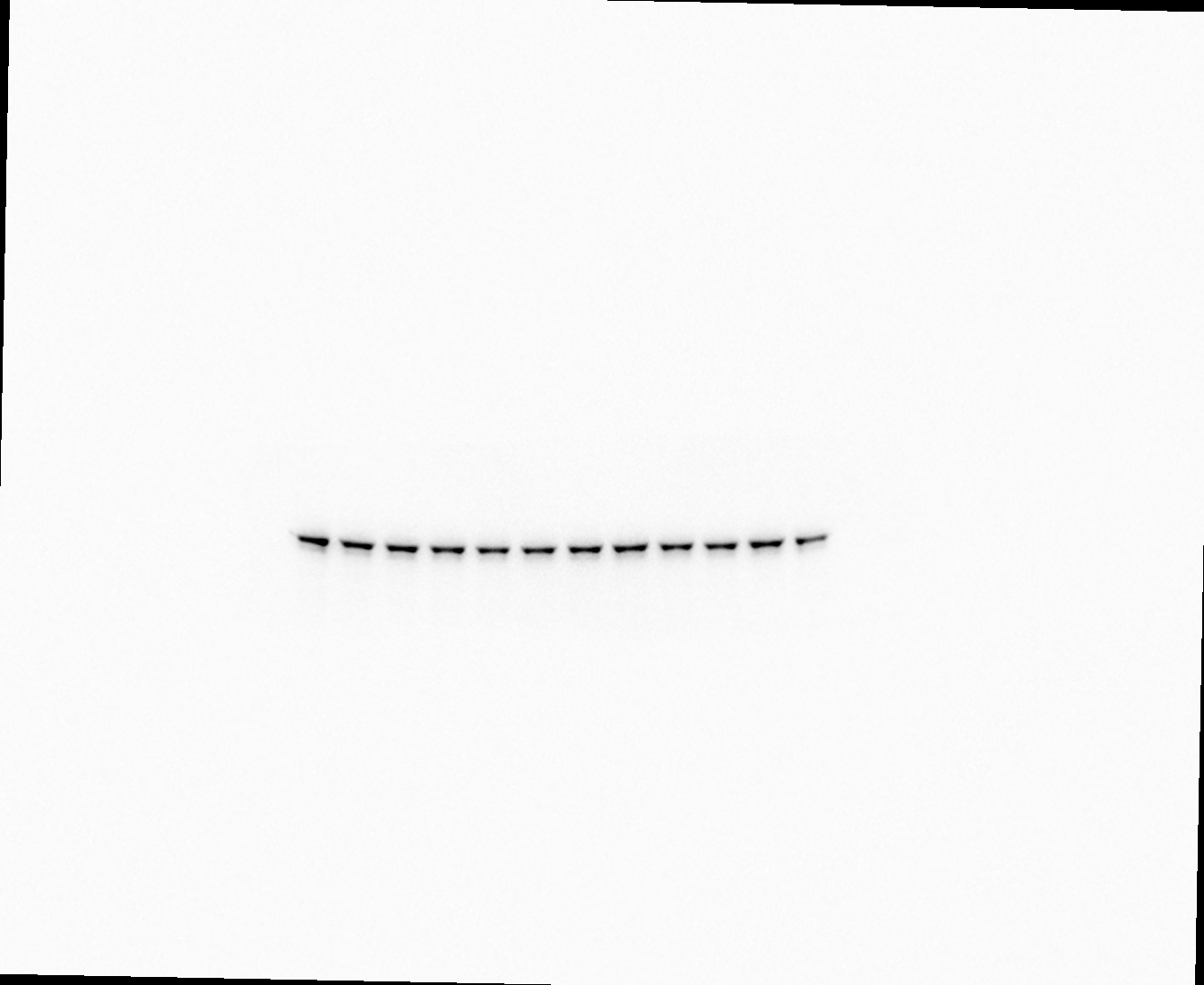


**Figure 4E**

**FOXL2**

**
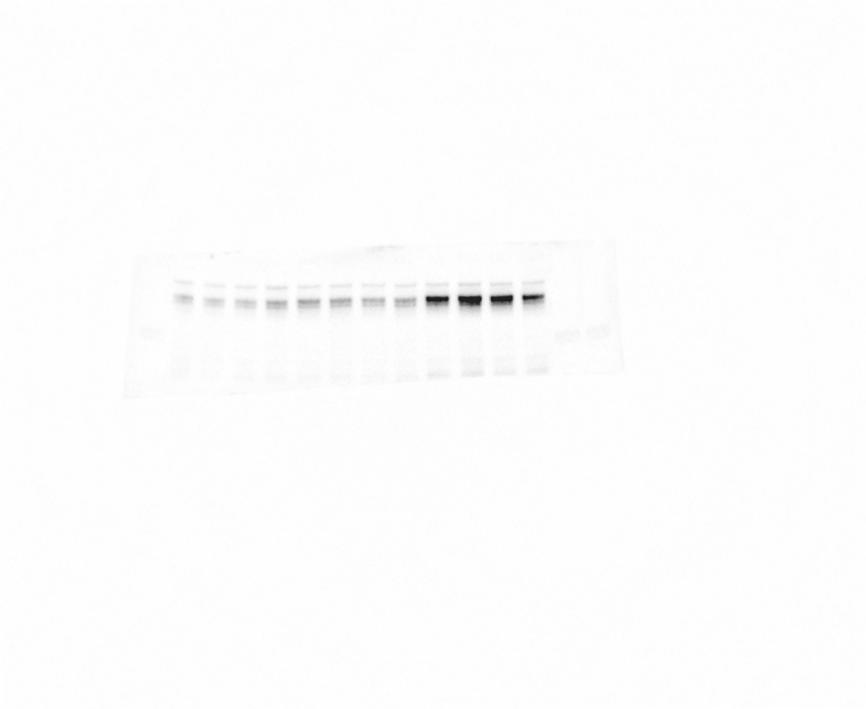
**

**β-actin**

**
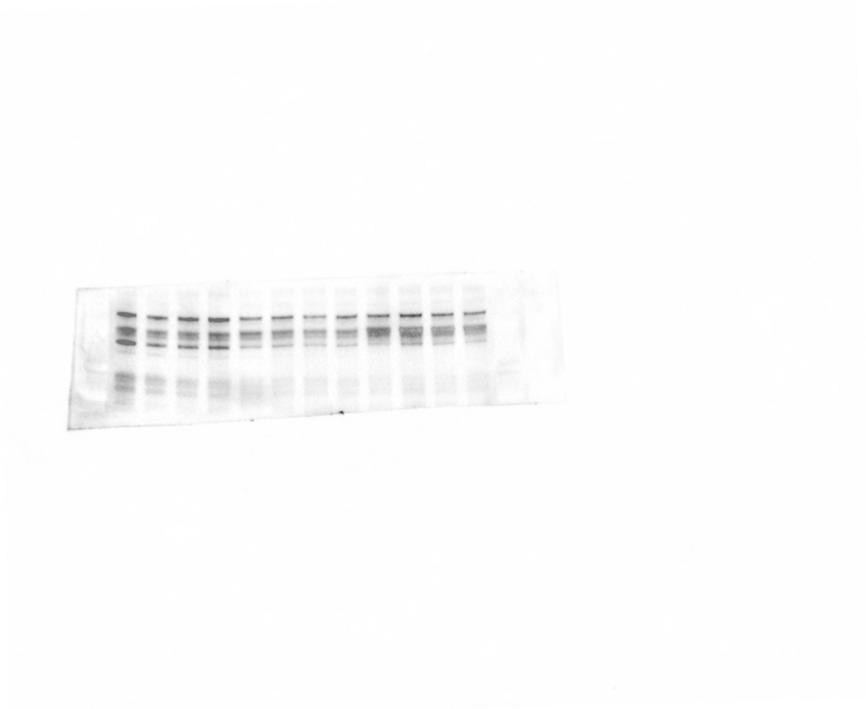
**

**Figure 5A**

**FOXL2**


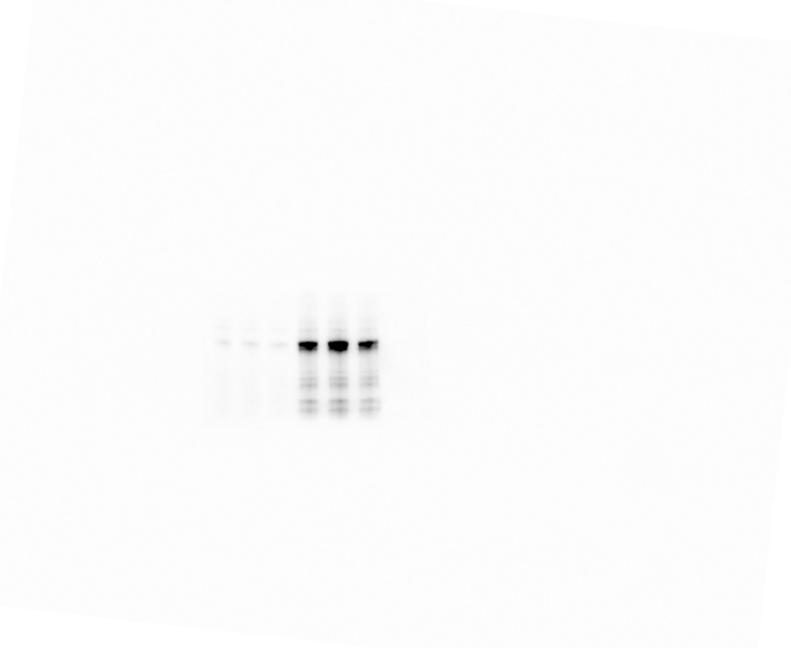


**StAR**


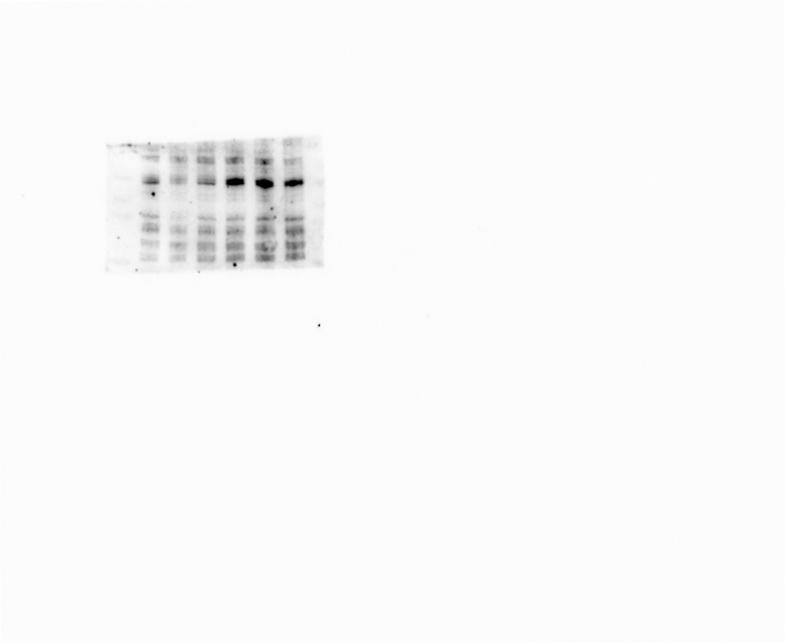


**CYP11A1**


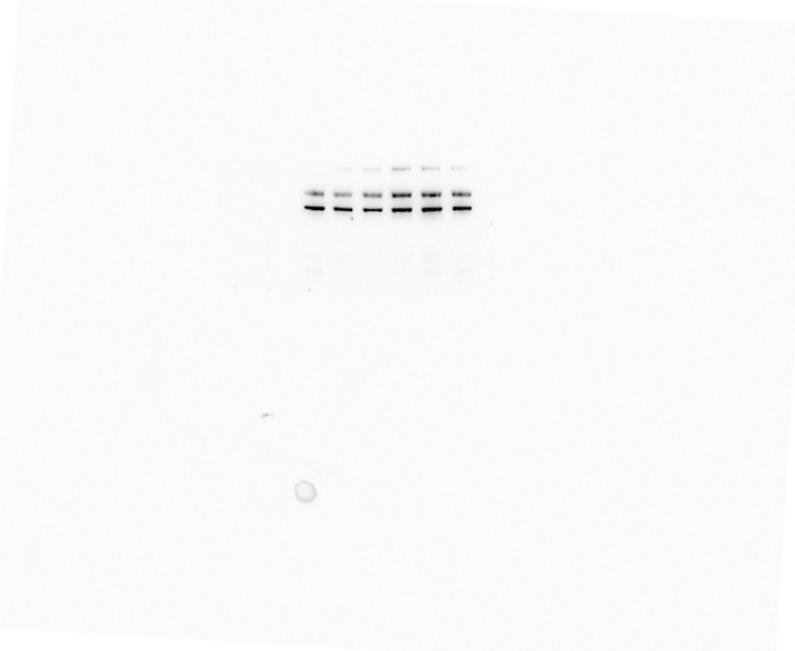


**CYP17A1**


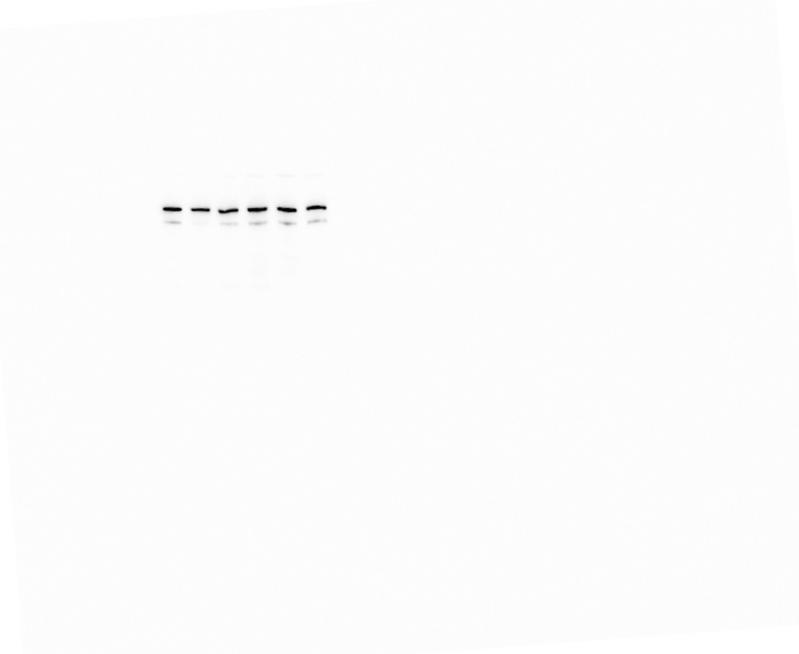


**CYP19A1**


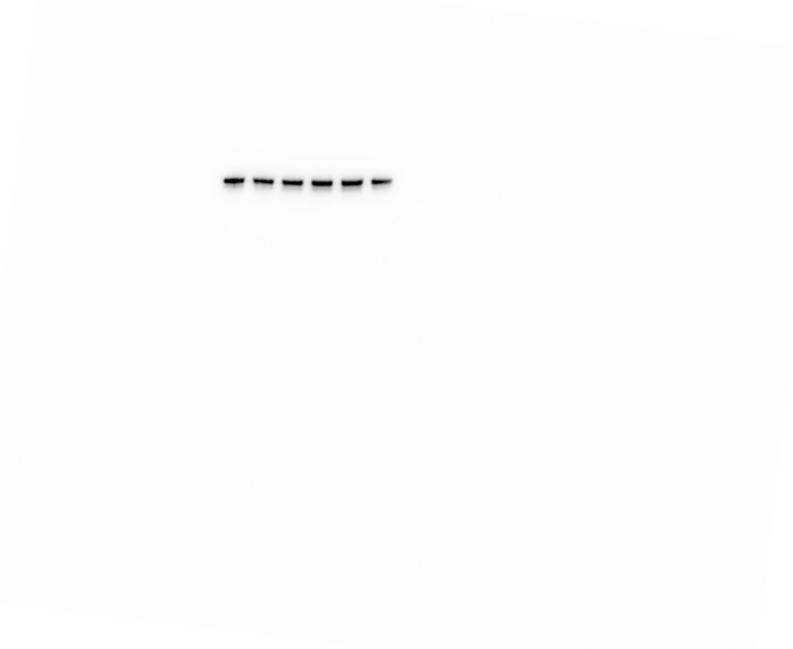


**β-actin**


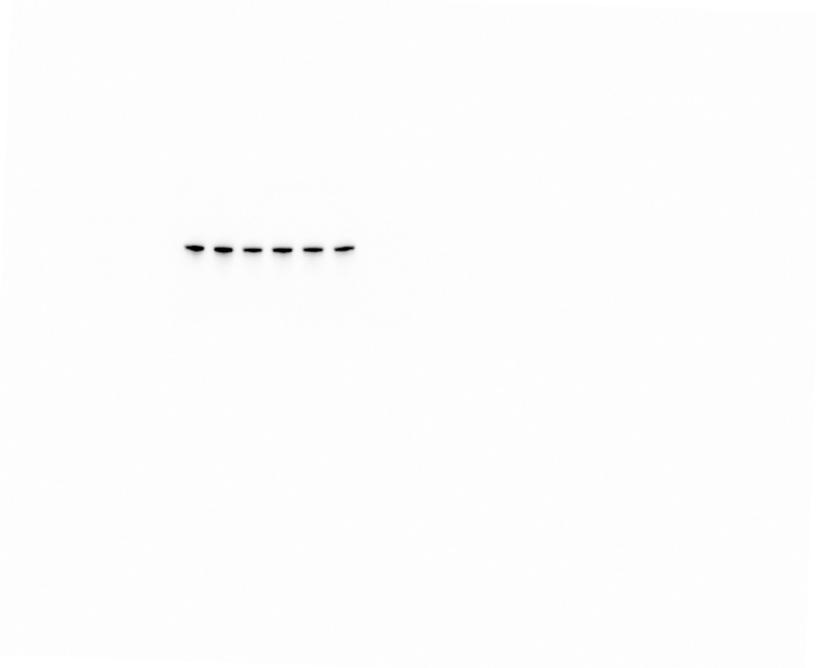


**Figure 5C**

**FOXL2**


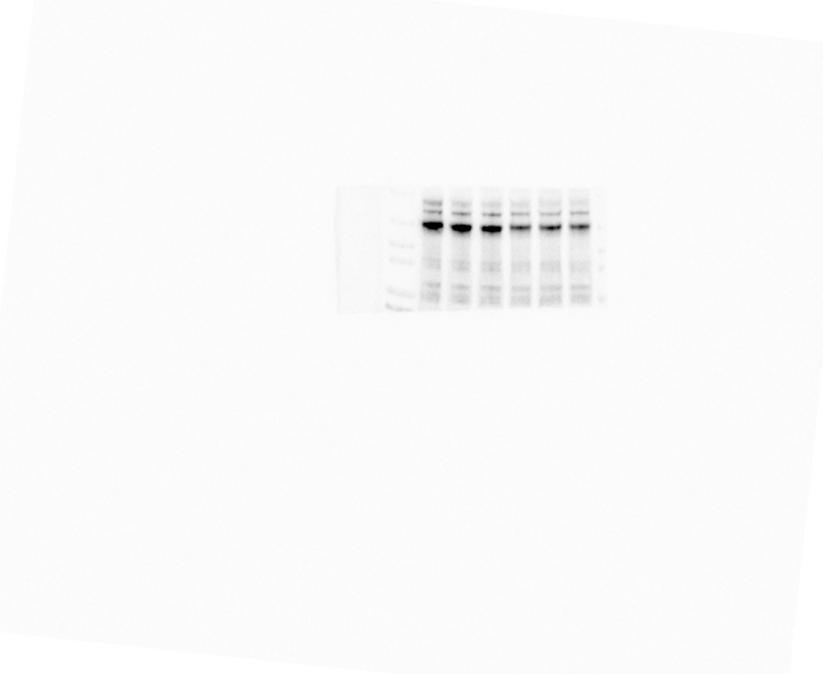


**StAR**


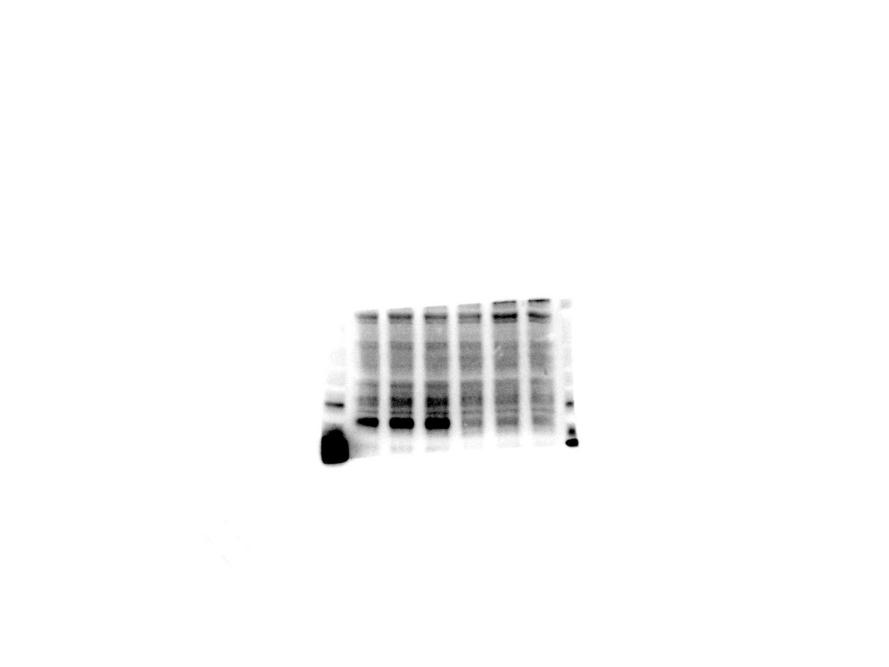


**CYP11A1**


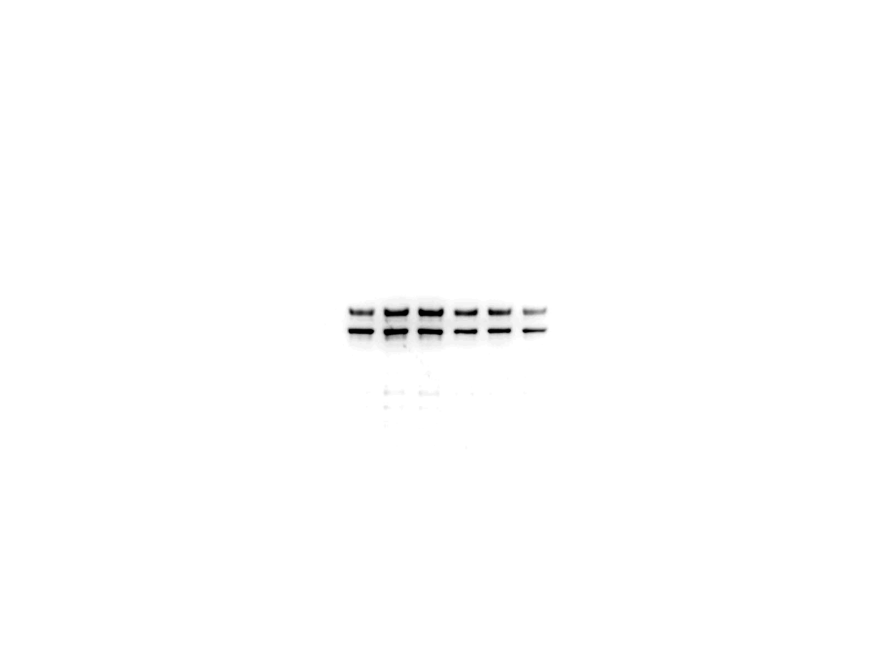


**CYP17A1**


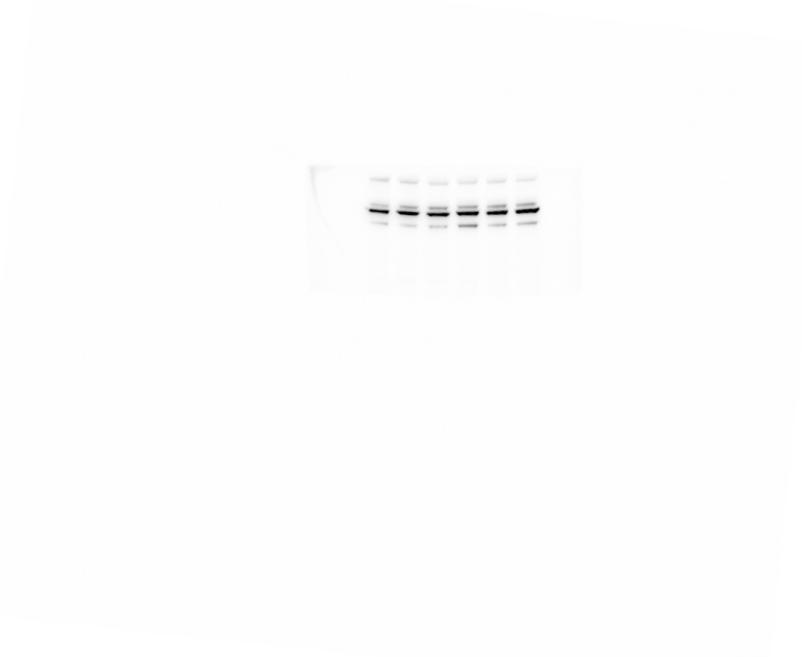


**CYP19A1**


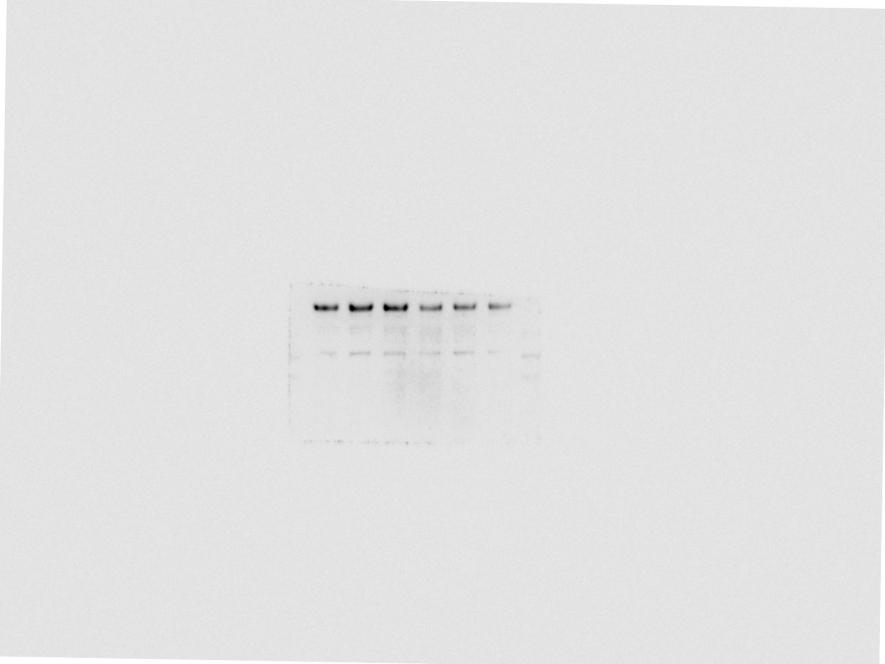


**β-actin**


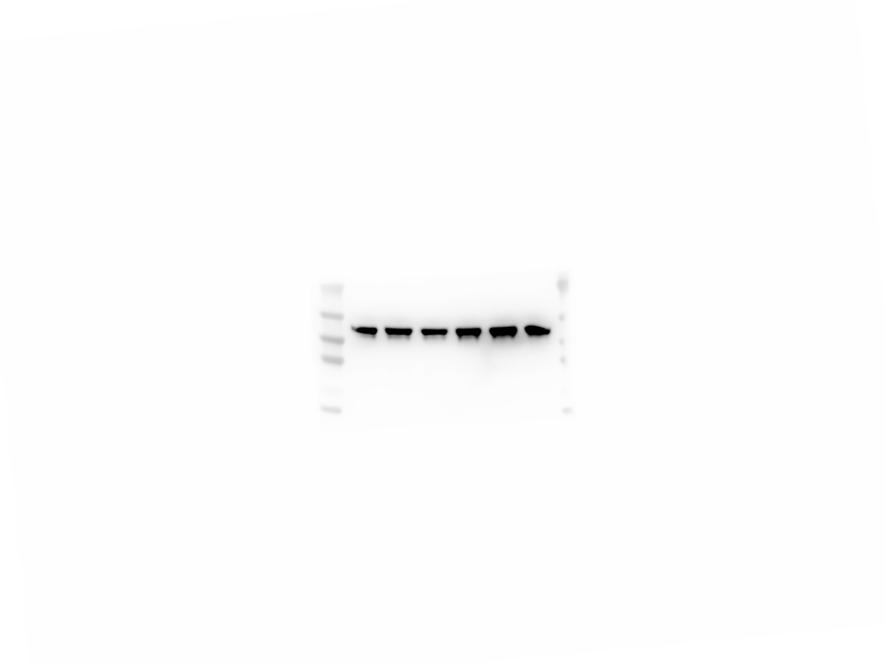


**Figure 5E**

**FOXL2**


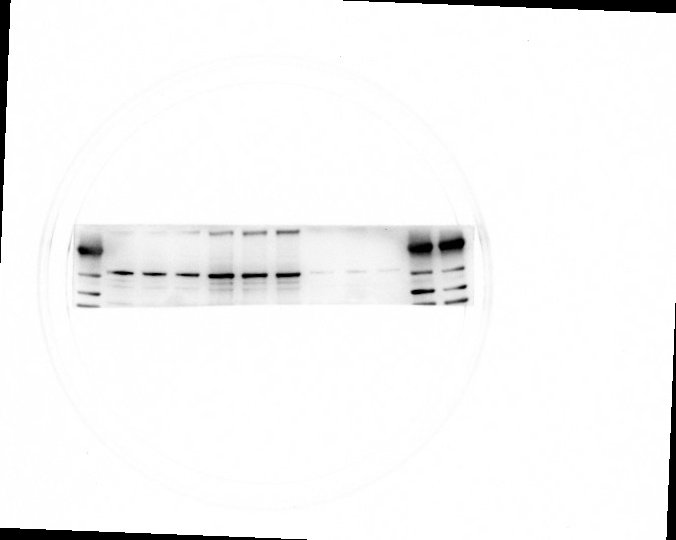


**StAR**


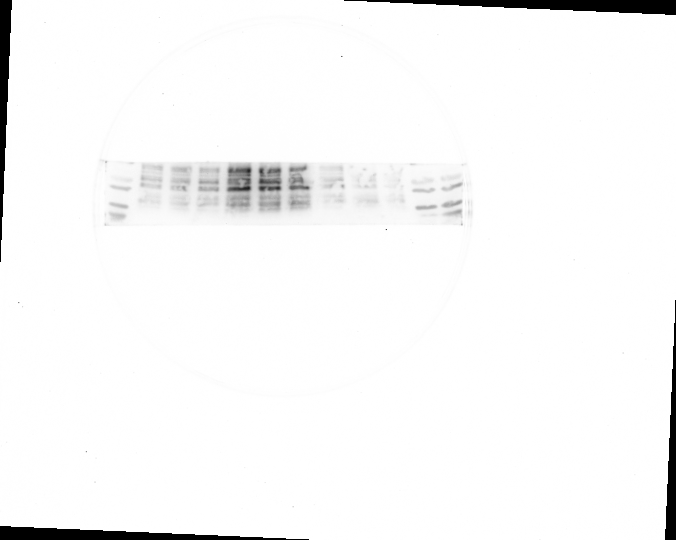


**CYP11A1**


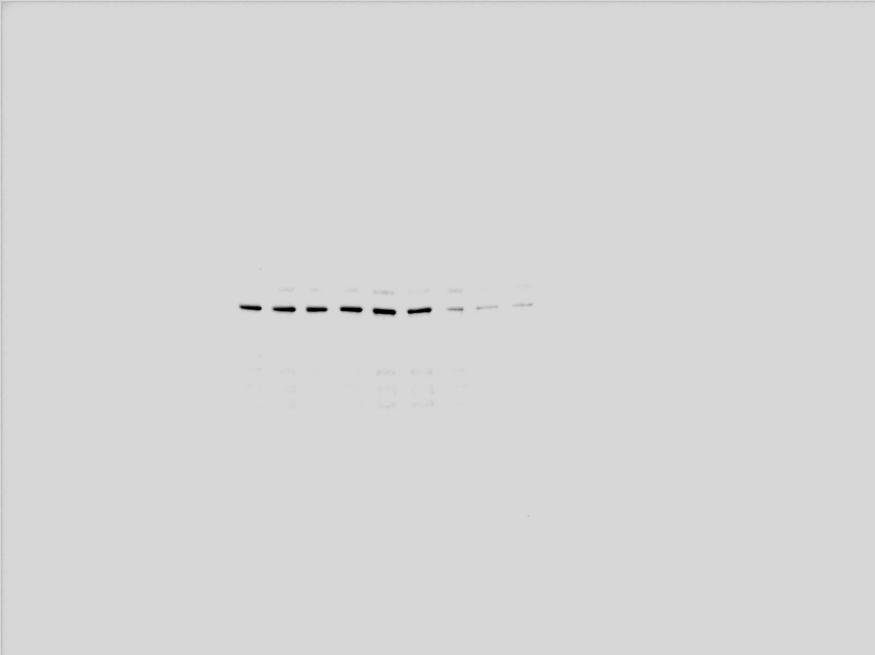


**CYP17A1**


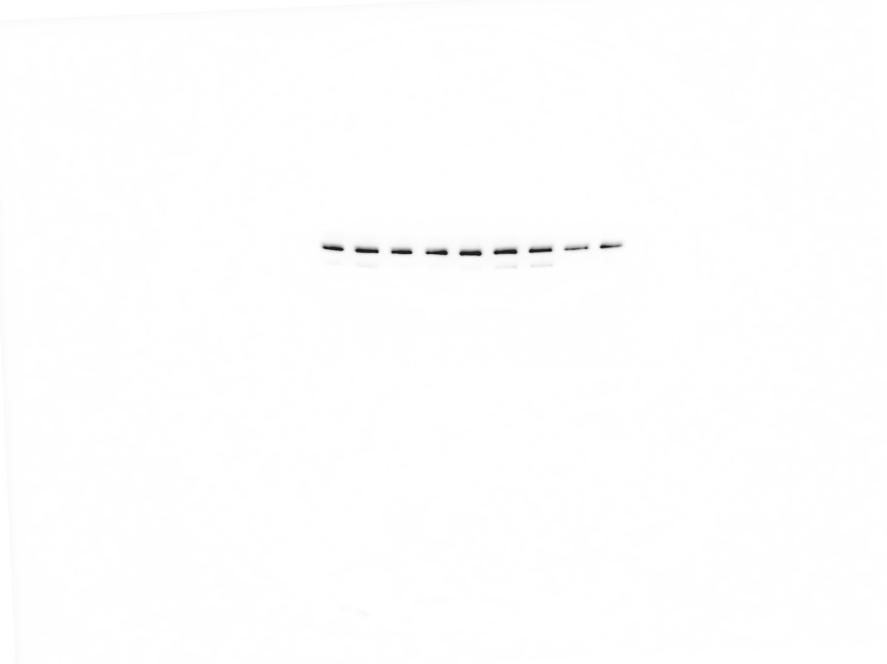


**CYP19A1**


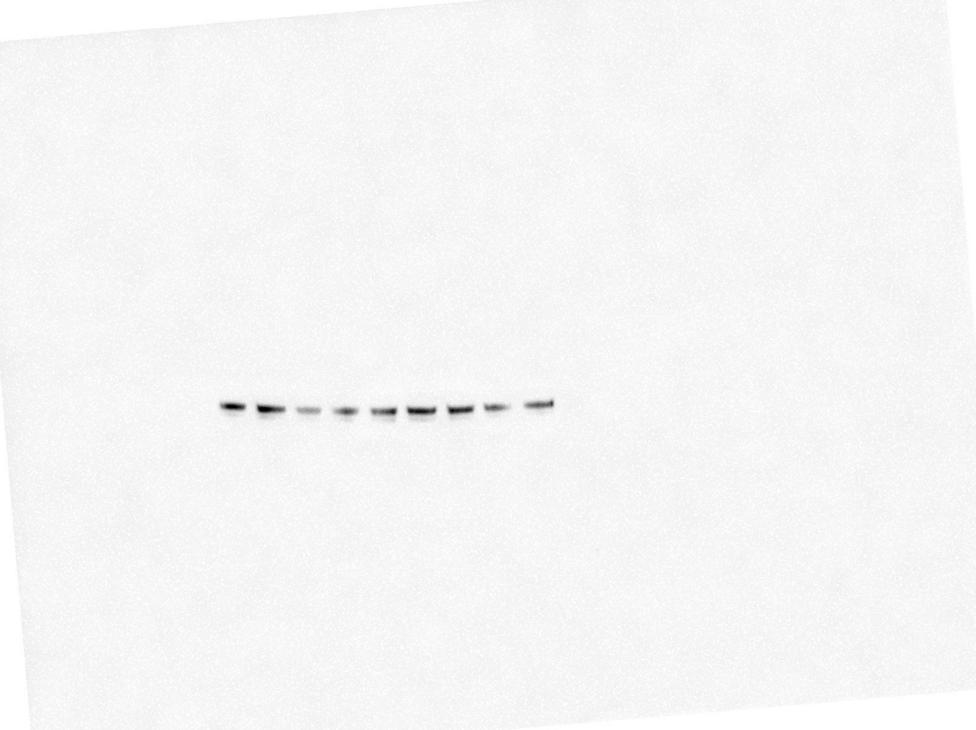


**β-actin**


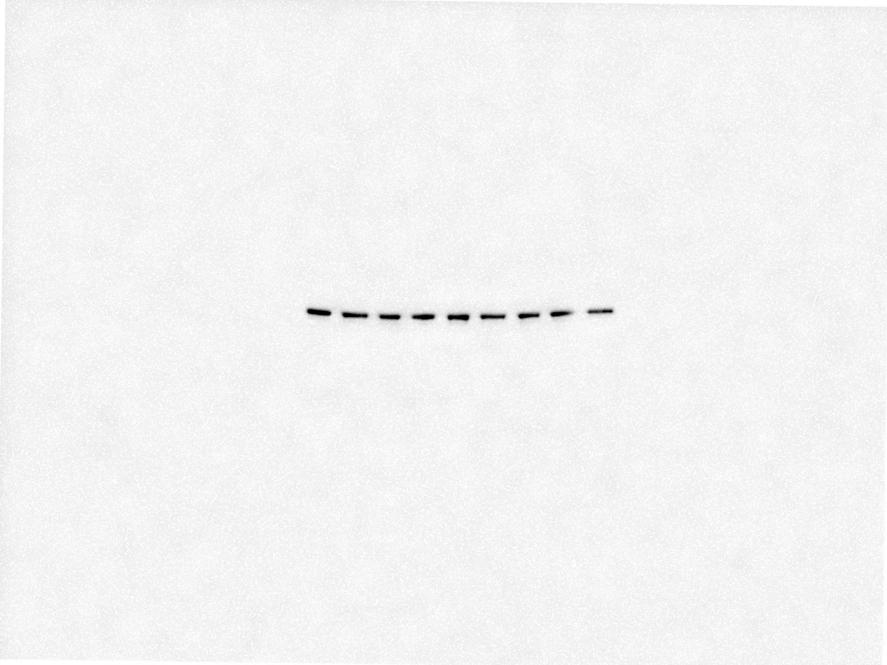


**Figure 6F**

**PLIN2**

**
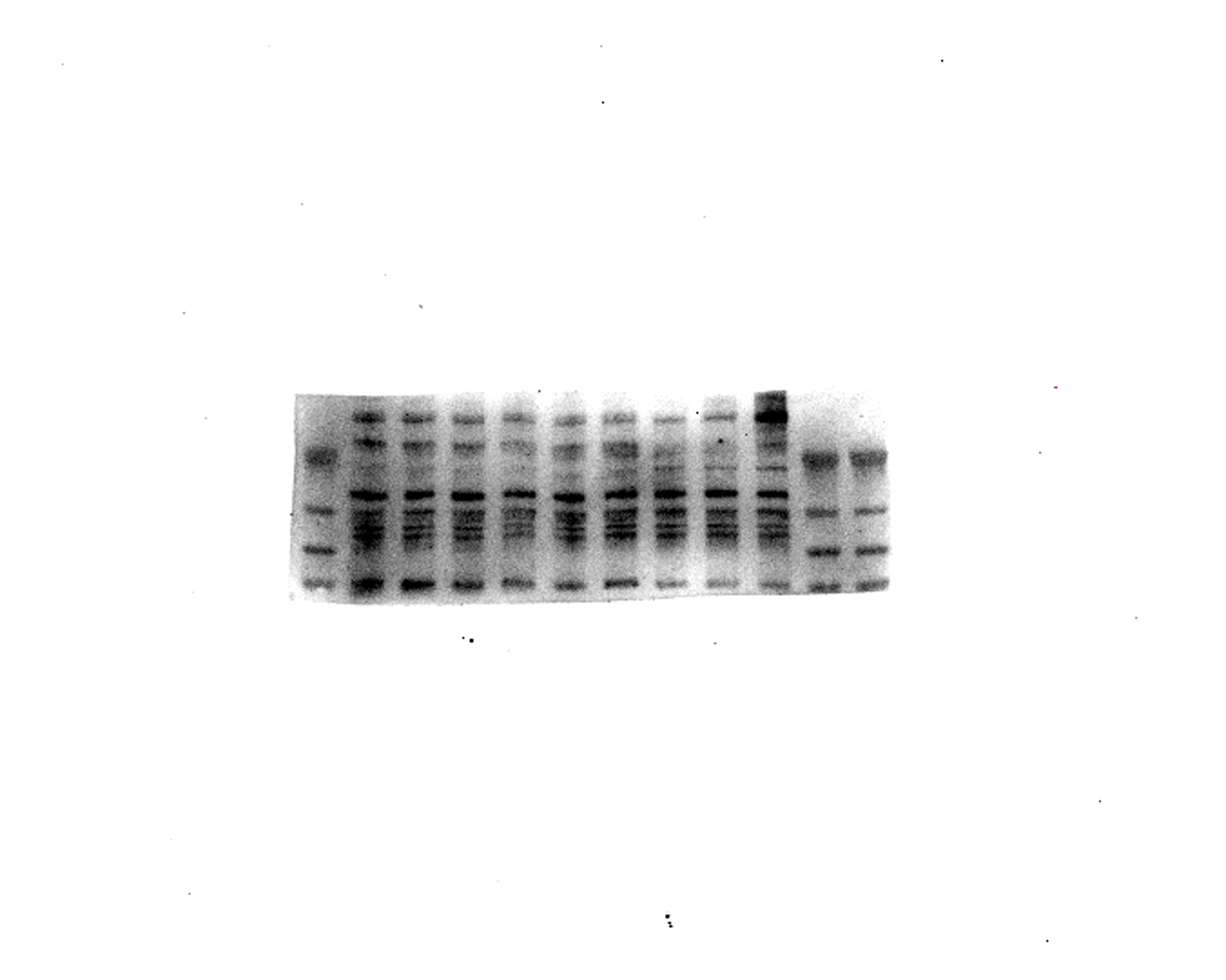
**

**β-actin**

**
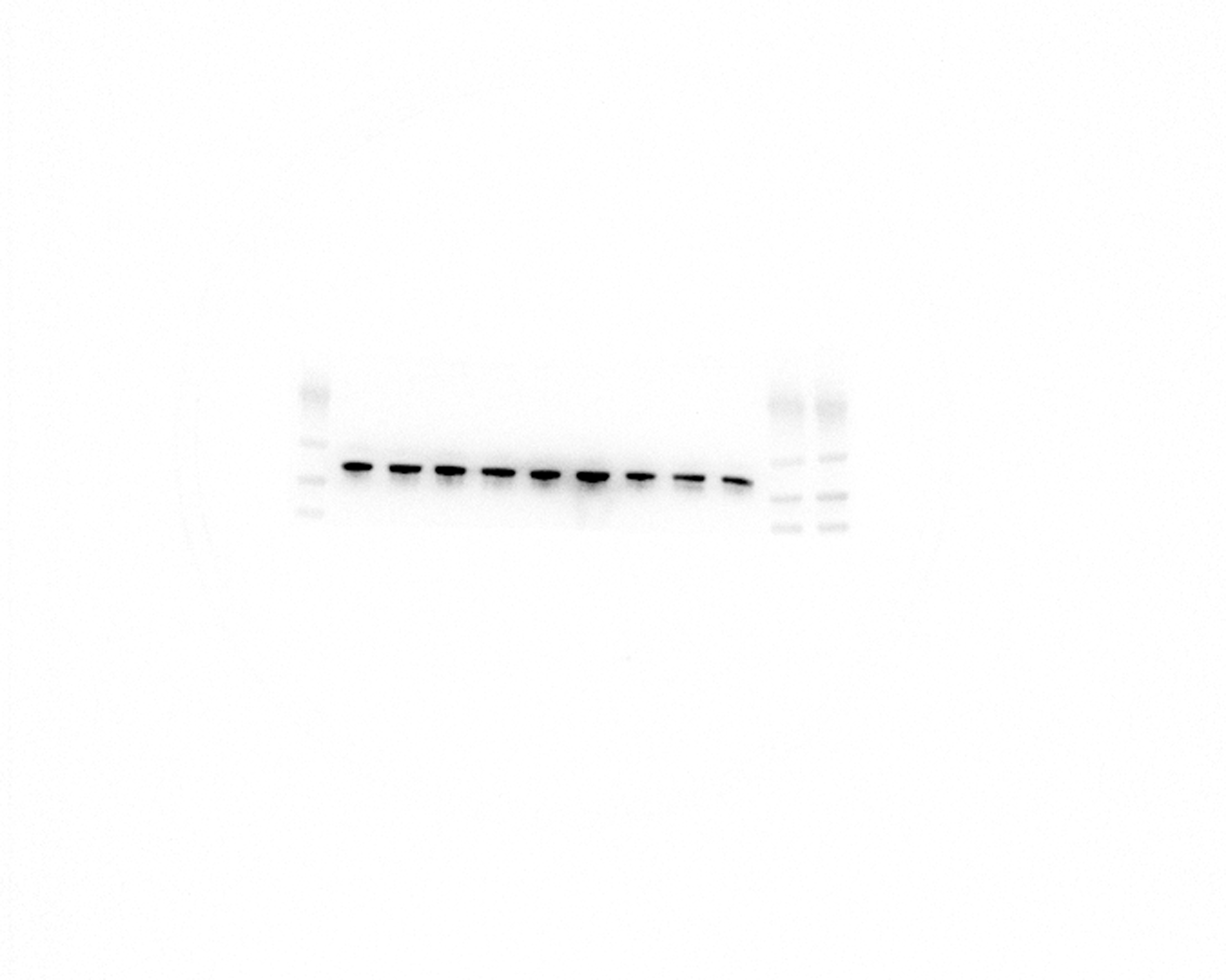
**

**Figure 7H**

**CPT2**

**
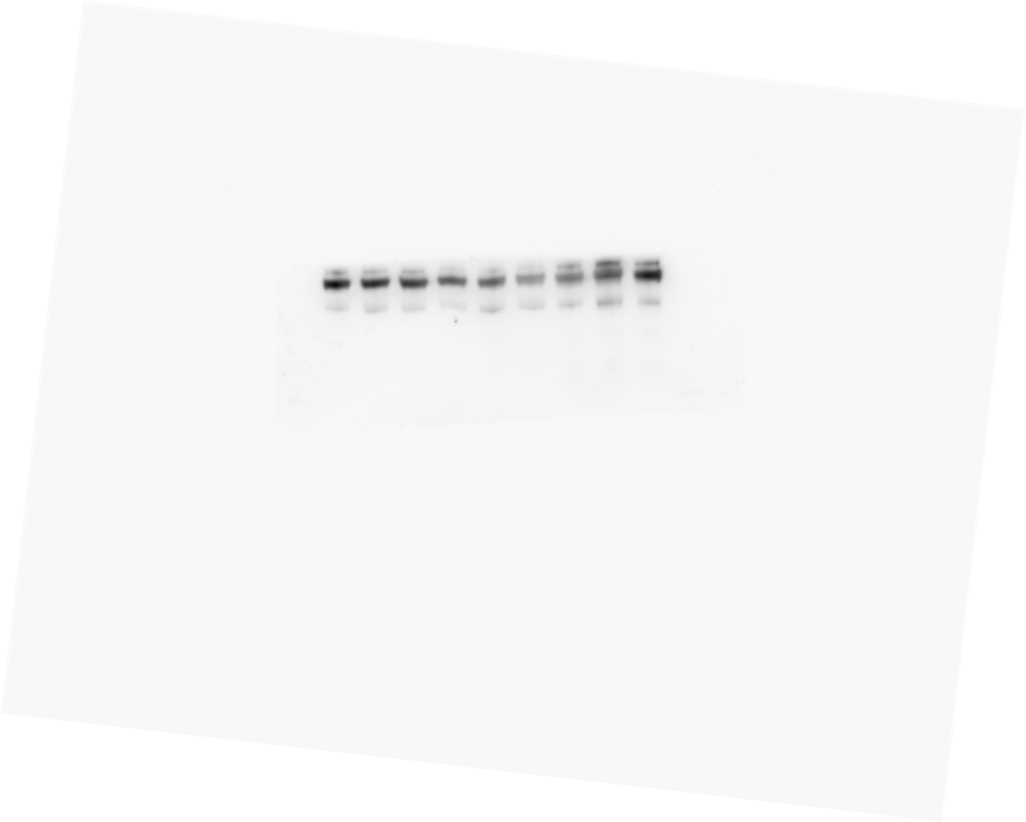
**

**ACSL4**

**
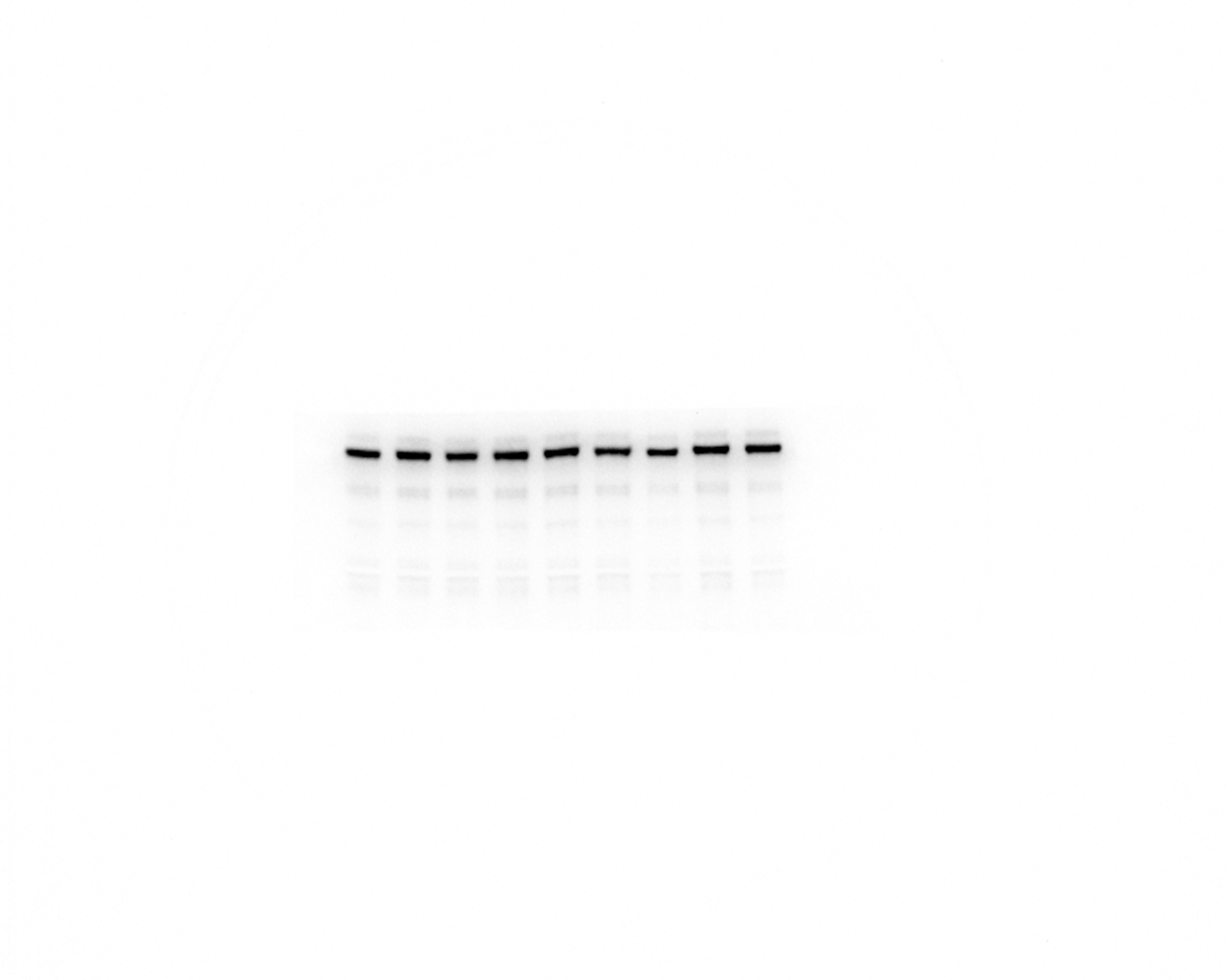
**

**β-actin**

**
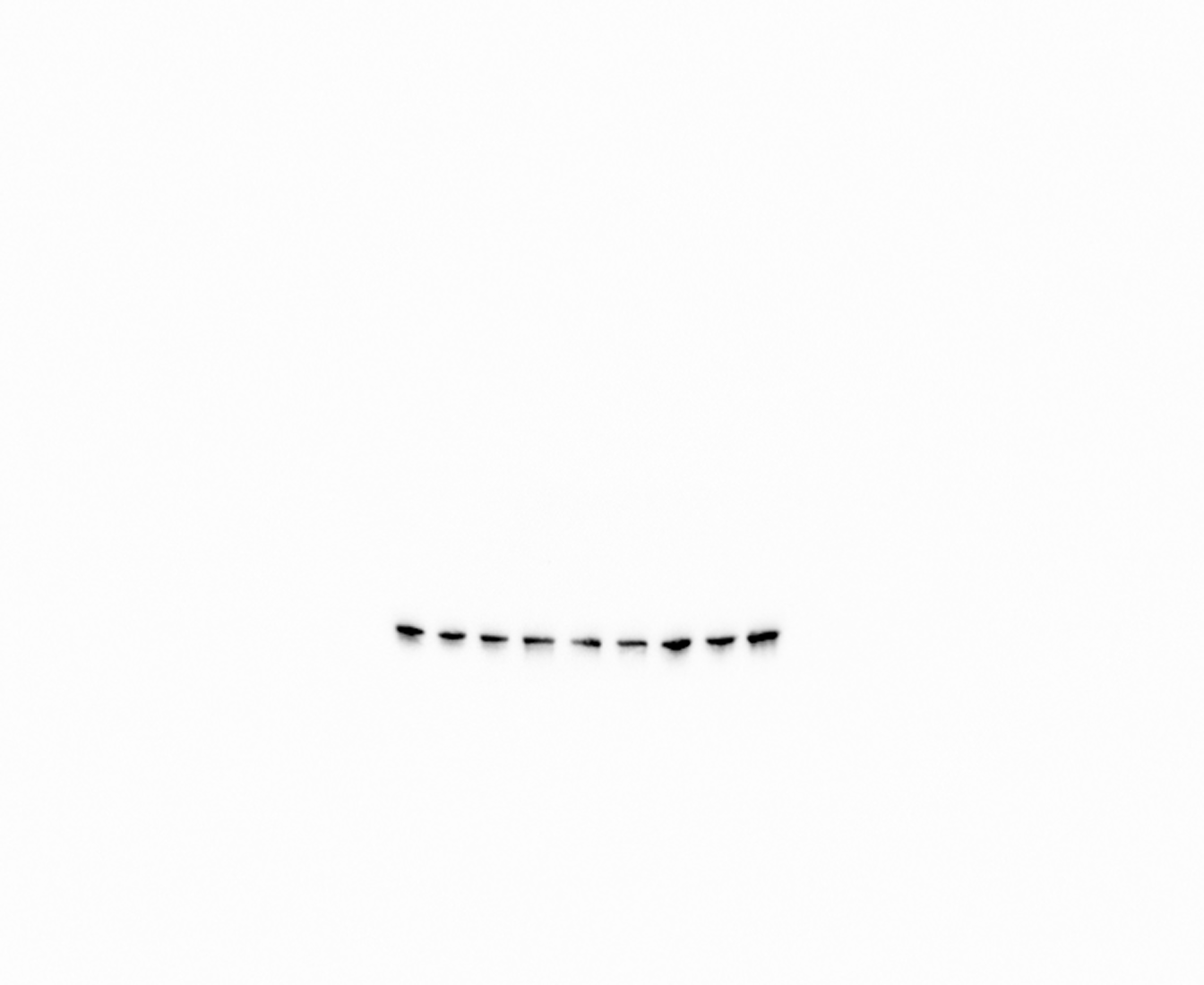
**

**Figure 7C**

**SOD1**

**
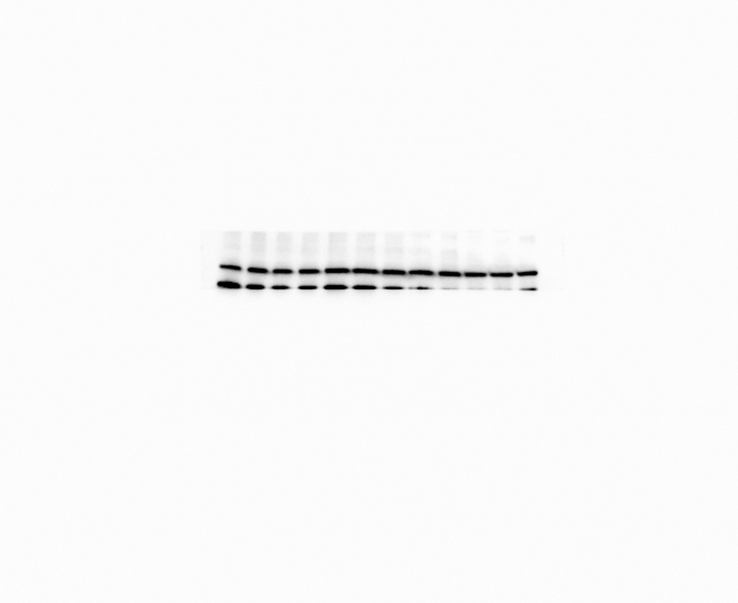
**

**SOD2**

**
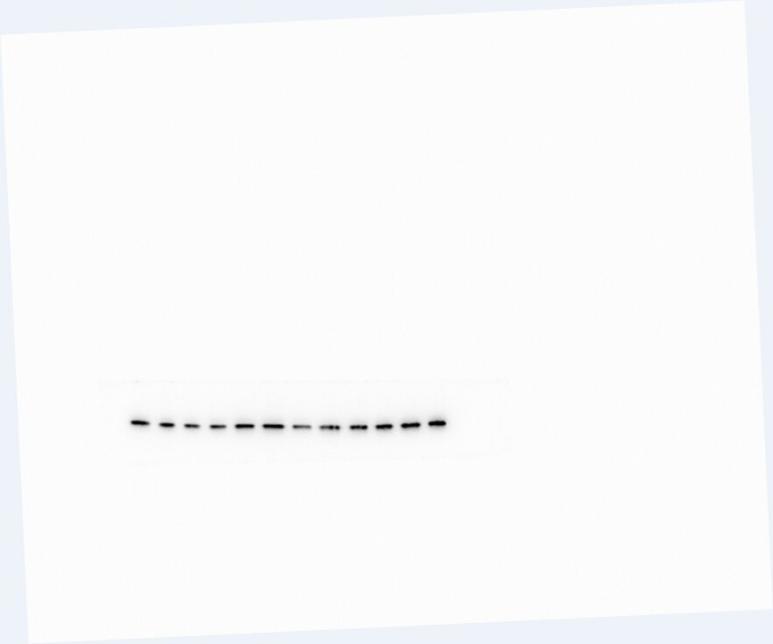
**

**GPX4**

**
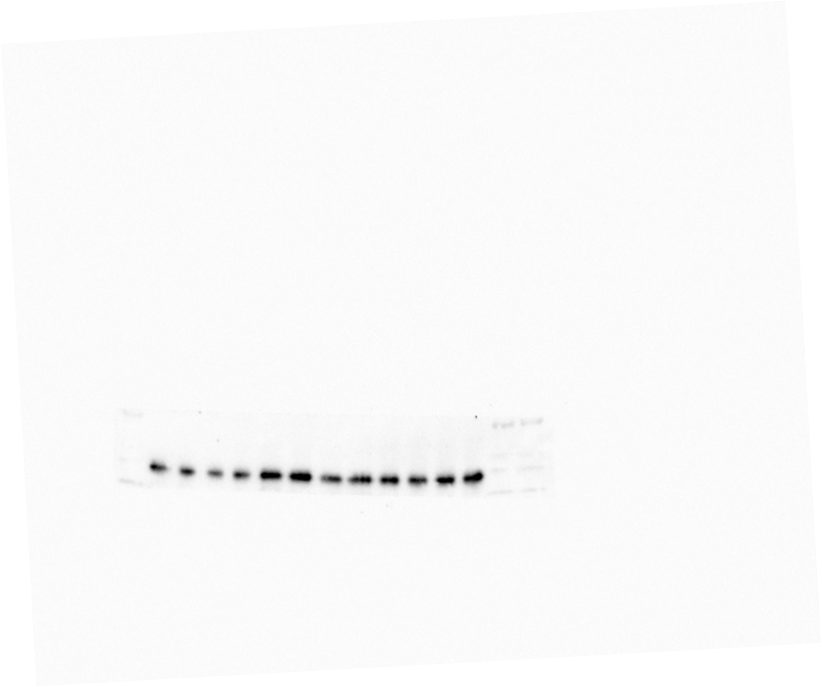
**

**CAT**

**
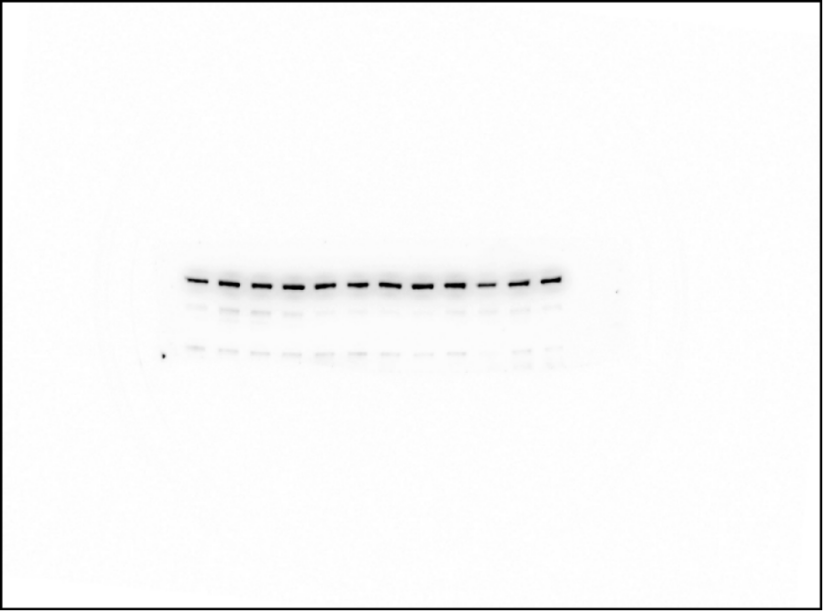
**

**actin**


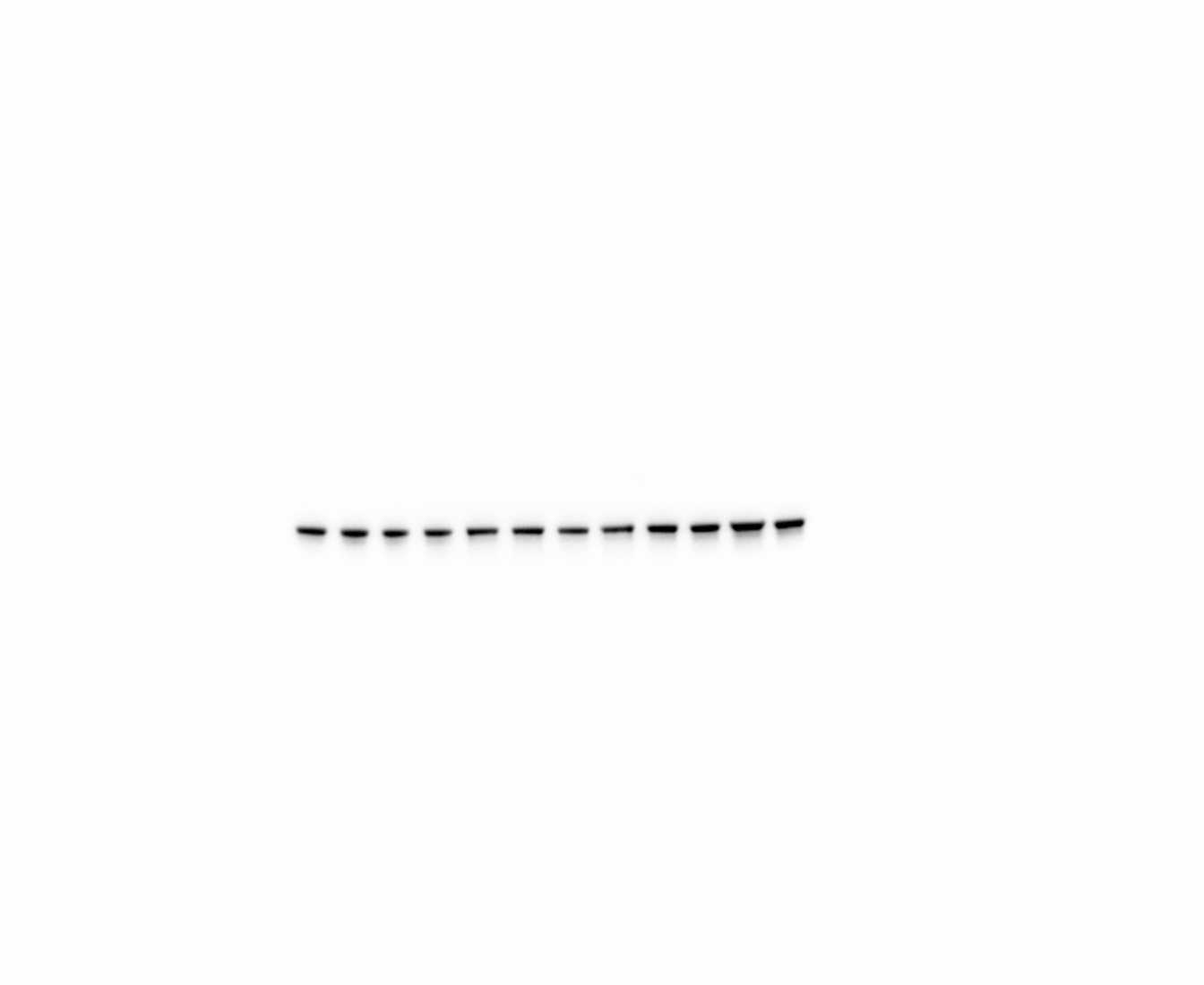

Supplement: Supplementary file 3 — Additional file 3. The full Western blot images. [file 40104_2025_1342_MOESM3_ESM.docx]
